# MetaMIS: a metagenomic microbial interaction simulator based on microbial community profiles

**DOI:** 10.1186/s12859-016-1359-0

**Published:** 2016-11-25

**Authors:** Grace Tzun-Wen Shaw, Yueh-Yang Pao, Daryi Wang

**Affiliations:** Biodiversity Research Center, Academia Sinica, Taipei, 115 Taiwan

**Keywords:** Metagenomics, Lotka-Volterra, Network dynamics

## Abstract

**Background:**

The complexity and dynamics of microbial communities are major factors in the ecology of a system. With the NGS technique, metagenomics data provides a new way to explore microbial interactions. Lotka-Volterra models, which have been widely used to infer animal interactions in dynamic systems, have recently been applied to the analysis of metagenomic data.

**Results:**

In this paper, we present the Lotka-Volterra model based tool, the **Meta**genomic **M**icrobial **I**nteracticon **S**imulator (MetaMIS), which is designed to analyze the time series data of microbial community profiles. MetaMIS first infers underlying microbial interactions from abundance tables for operational taxonomic units (OTUs) and then interprets interaction networks using the Lotka-Volterra model. We also embed a Bray-Curtis dissimilarity method in MetaMIS in order to evaluate the similarity to biological reality. MetaMIS is designed to tolerate a high level of missing data, and can estimate interaction information without the influence of rare microbes. For each interaction network, MetaMIS systematically examines interaction patterns (such as mutualism or competition) and refines the biotic role within microbes. As a case study, we collect a human male fecal microbiome and show that *Micrococcaceae*, a relatively low abundance OTU, is highly connected with 13 dominant OTUs and seems to play a critical role. MetaMIS is able to organize multiple interaction networks into a consensus network for comparative studies; thus we as a case study have also identified a consensus interaction network between female and male fecal microbiomes.

**Conclusions:**

MetaMIS provides an efficient and user-friendly platform that may reveal new insights into metagenomics data. MetaMIS is freely available at: https://sourceforge.net/projects/metamis/.

**Electronic supplementary material:**

The online version of this article (doi:10.1186/s12859-016-1359-0) contains supplementary material, which is available to authorized users.

## Background

Propelled by 16S ribosomal RNA (rRNA) sequencing technologies, there has recently been a growing interest in characterizing the role of complex microbial communities in a diverse ecosystem. As a result, an increasing number of samples from marine, soil [1], animal feces, and mammalian gut microflora [2] has been placed in the public domain. Studies have shown that health status, habitat types, and external perturbations are some of the key factors that can change a microbial community in specific ecosystem niches. For instance, the human gut harbors a vast number of microbial species, and imbalances in the intestinal microbiome have been linked with such chronic diseases as obesity [3], inflammatory bowel disease [4], and type 2 diabetes [5]. Marine microbes sensitive to changing climates also play an important role in ocean feedback, being associated with such phenomena as surface warming, ice melting, and acidification, as well as climate change [6]. From the human gut to global oceans, metagenomic studies offer new insights into compositional stability. However, a deeper investigation into microbial interactions, including mutualism (+/+), competition (−/−), parasitism or predation (+/−), commensalism (+/0), and amensalism (−/0), as reviewed by Faust and Raes [7], is required to fill gaps in understanding of the relationships between microbial communities and hosts or environments. Fortunately, with recent efforts on bioinformatics, some computational approaches using metagenomic data have suggested that association networking and modeling show promise as tools for characterizing multilevel interactions and elucidating the temporal dynamics exhibited by microbial communities.

Discerning the full extent of the web of microbial interactions is a difficult task. The conventional approach is to observe the growth behavior in mixed cultures of only a very few microorganisms [8]. Recently, high-throughput interaction inference approaches, such as Sparse Correlations for Compositional data (SparCC) [9], the Learning Interactions from MIcrobial Time Series (LIMITS) algorithm [10], co-occurrence networks [11], the SParse InversE Covariance estimation for Ecological ASsociation Inference (SPIEC-EASI) [12], and the Rule-based Microbial Network (RMN) algorithm [13], have been proposed for modeling microscale dynamics using 16S rRNA marker gene sequences. These approaches may be roughly divided into two categories. Correlation-based methods, including SparCC [9] and co-occurrence networks [11], aim to develop algorithms that combine correlation methods in order to decipher highly dependent temporal microbial communities that have usually proved refractory to classical correlation analysis. Although correlation is straightforward and easy to conduct, it nevertheless does not seem to be a proper measure of species interactions, and is limited to inferring non-directional interactions [11, 12]. Modeling-centered approaches, on the other hand, including the LIMITS [10], SPIEC-EASI [12], and RMN [13] algorithms, rest on special biological assumptions and statistical techniques, and usually employ a combined strategy in order to infer microbial interactions. LIMITS, for instance, combines a spare linear regression with a bootstrapping strategy in order to incorporate interactive relations iteratively into an interaction network [10]. SPIEC-EASI assumes the underlying ecological association network to be sparse and accordingly relies on sparse inverse covariance selection and a neighborhood selection strategy to reconstruct a non-directional interaction network [12]. The RMN algorithm bypasses the NP-hard problem of finding a network with the optimum number of interactions and proceeds directly to the construction of a triplet subnetwork in which the triplet has a convergent recipient that is repressed by one interaction and simultaneously activated by another [13].

Although much work has been done to date, more study is necessary to ascertain the effects of inferring a direct comprehensive interaction network on a variety of network inference methods. Among the methods mentioned above, the LIMITS and RMN algorithms offer a more sound theoretical basis for inferring a direct interaction network, but cause complications for the comprehensive inference of an interaction network. To that end, early attempts at exploiting a direct comprehensive interaction network from microorganisms have been successfully conducted using the Lotka-Volterra model, as first proposed by Jansen [14] and commonly employed by ecologists, which can describe systematically a dynamic trophic web of more than two macro-organism populations. When applied to a metagenomic abundance generator, the Lotka-Volterra model can successfully generate a simulated microbial community given a set of known interspecies interactions [10, 11, 15]. When applied to simulating microbial interactions, recent studies on lake ecosystem [16], mouse intestine [17] and cheese-making environments [18] have shown that Lotka-Volterra equations can quantify microbial interactions and successfully predict microbiome temporal dynamics. Moreover, a previous study has demonstrated that the distribution of simulated interaction pairs in an ecological system can be used to predict microbiome stability. For instance, a cooperative network of microbes (i.e., one characterized by mutualism) is often unstable, while a higher proportion of competitive interaction pairs (−/−) helps the host to maintain a stable microbial community [15]. Thus the Lotka-Volterra model, which, as mentioned, is commonly used to illustrate the dynamics of macro-ecologcal communities, may shed light on the complex world of microbial communities.

Detecting and investigating the structure of interactions in microbial ecosystems is, then, absolutely critical, but the reconstruction of ecosystem-wide association networks using the Lotka-Volterra model is far from straightforward. Here we present a stand-alone tool called MetaMI that aims to facilitate the systematic inference of microbial interactions. The characteristics of MetaMIS are as follows. (i) User-friendly interface: we have constructed an easy-to-use graphic user interface (GUI) for scientists, even those who lack programing skills, to infer microbial interactions. (ii) Network topological visualization: MetaMIS offers two ways to visualize the inferred microbial interactions. If there are N microbes in an interaction network, a general view includes the minimum number of interaction pairs to describe N microbes. A specific view of a single microbe takes into account the interactive behaviors of one microbe in relation to all others. (iii) Maximal detection of rare population: while rare species are usually regarded as noise in most quantitative ecological analysis, MetaMIS provides the opportunity to evaluate the fitness of each rare species in a microbial system by means of an abundance-ranking strategy. (iv) Consensus network: MetaMIS is able to unify multiple interaction networks into a confident network.

To provide a user friendly interface, MetaMIS was designed to accept microbial abundance profiles in regular text format on both Mac and Windows (64-bit) platforms. MetaMIS has been tested using a human male intestinal microbiota dataset composed of 317 time points and 92 microbes at the family level and produced 27 prediction models in around 5 min on a current desktop computer. MetaMIS generates outputs in several formats that can be used with other popular network visualization software, such as Gephi [19] and Cytoscape [20]. The central purpose of MetaMIS is to provide clues about the interactions among microbes and about specific microbes in a microbial community. To our knowledge, no similar tool is available. MetaMIS is free to the public and can be accessed at a public IP address space without any login requirement: https://sourceforge.net/projects/metamis/.

## Implementation

### MetaMIS: overview

**Fig. 1:**
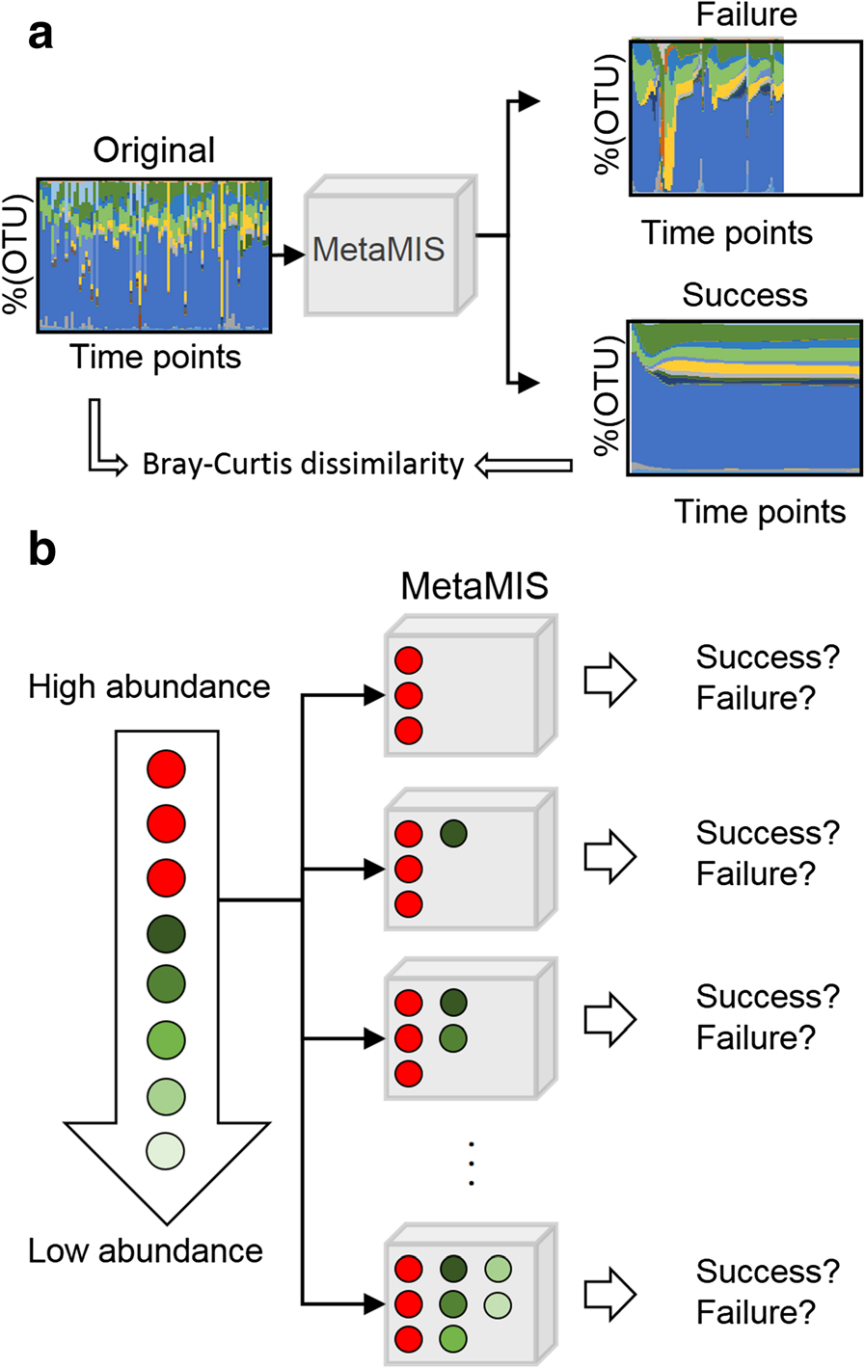
The rationale behind MetaMIS. **a** The input of MetaMIS consists of microbial abundance profiles, and after its implementation there are two possible outcomes, success or failure of the interaction network. **b** In a microbial community, abundance-ranking OTUs appeared sequentially in different network

**Fig. 2:**
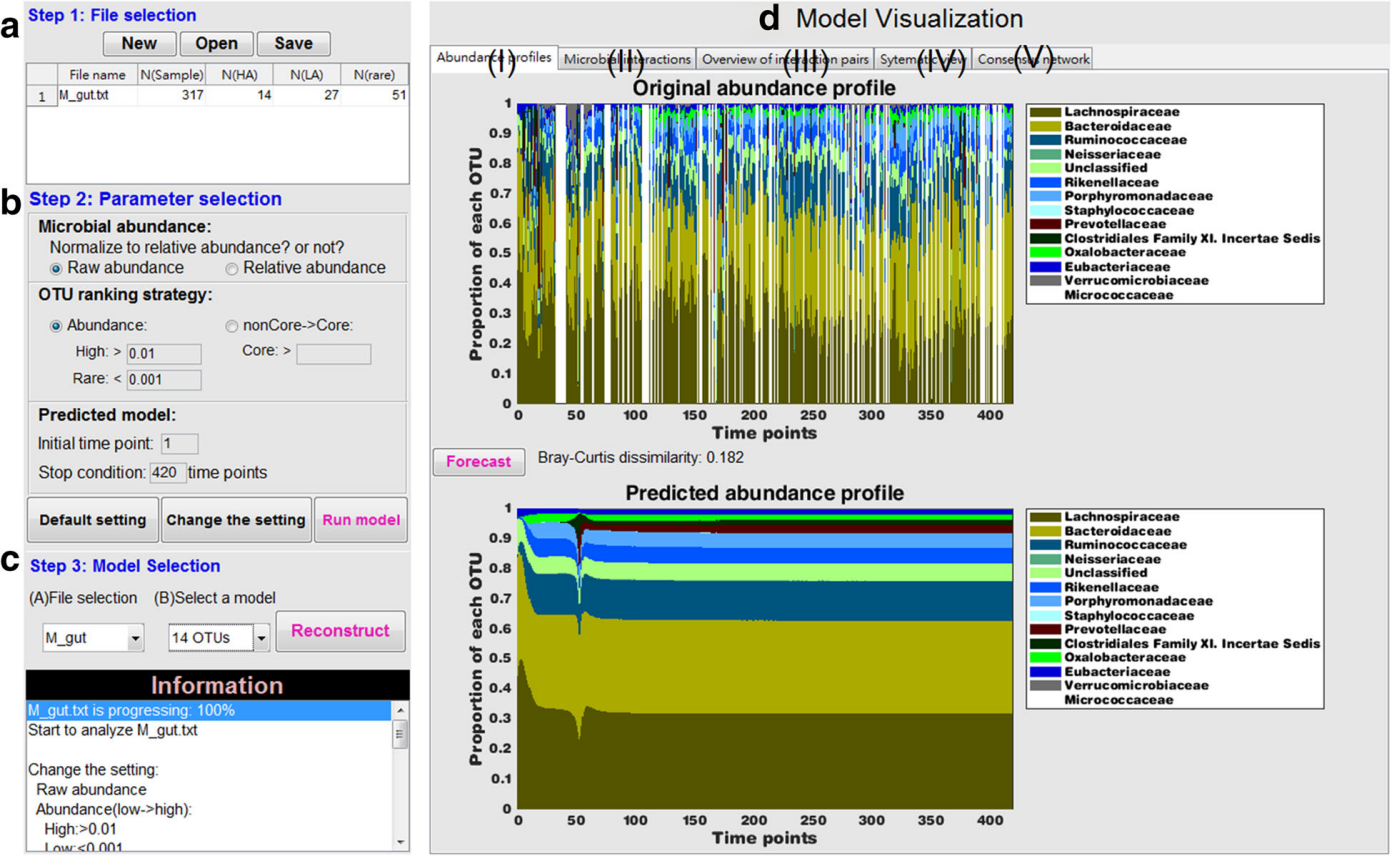
The interface of MetaMIS. A typical analytic workflow proceeds through four steps: (**a**) uploading formulated data file(s), (**b**) specifying the parameters, (**c**) performing the calculations for the network, and (**d**) visualizing the outputs, which comprise five panels, (I) to (V). See Fig. 3 for a detailed description of these panels

**Fig. 3:**
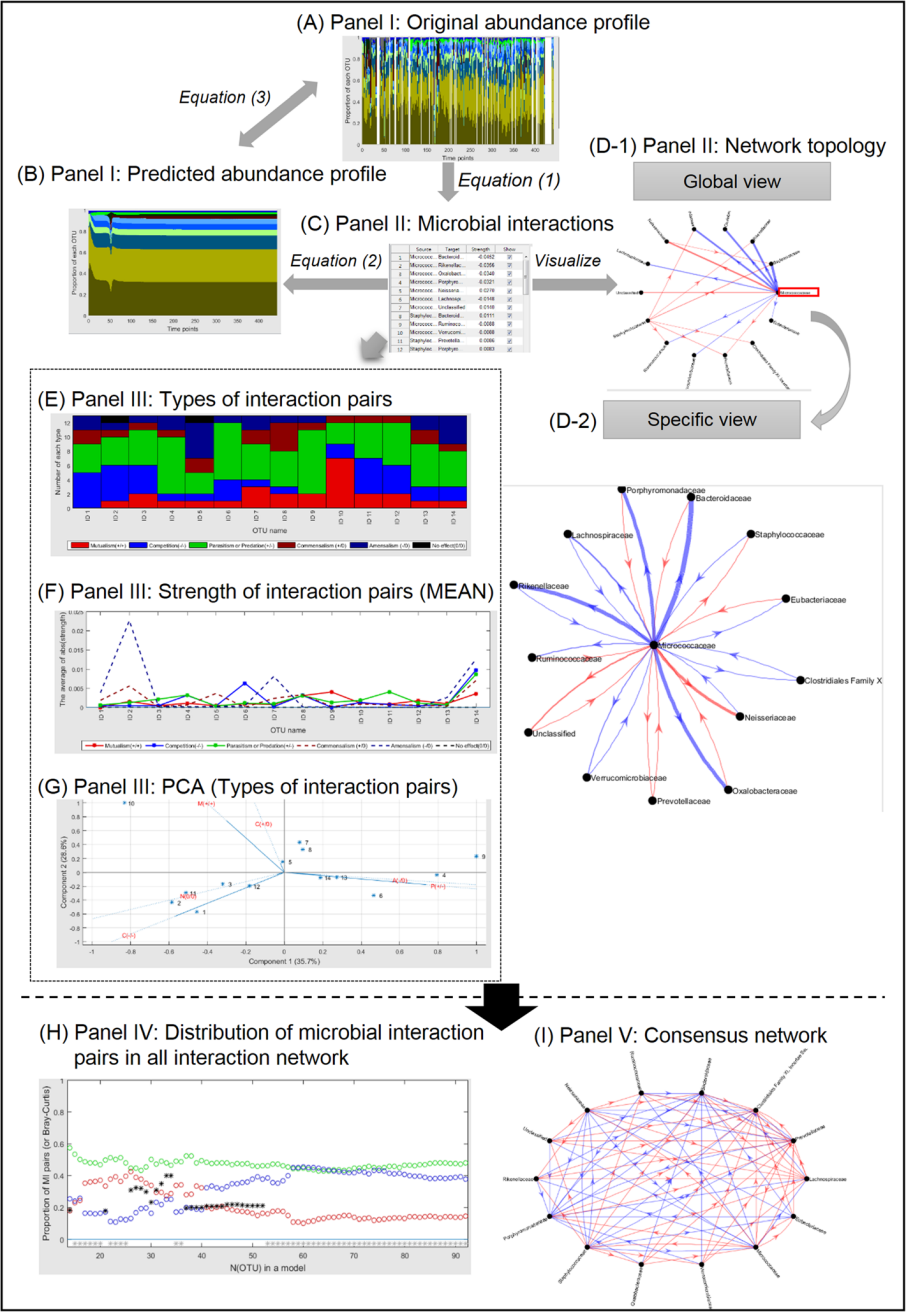
The analytic schema of MetaMIS. *Panel I* contains the original (**a**) and predicted (**b**) abundance profiles. Inferred microbial interactions are displayed in tabular form (**c**) and topologically (**d**), as shown by the global (**D-1**) and specific views (**D-2**) in *Panel II. Panel III* summarizes the distribution of interaction patterns (**e**) and their interactive strength (**f**) for each microbe. The PCA plot is intended to help users to identify key microbes (**g**). *Panel IV* provides a systematic diagram (**h**) to monitor and compare the performance from diverse interaction networks. *Panel V* displays a consensus network (**i**) in which interactions have more consensus directions among interaction networks

The central organizing metaphor of MetaMIS is the construction of microbial interaction networks, with microbial members, i.e., operational taxonomic units (OTUs). The network is presented with nodes and directed edges, in which nodes are OTUs and directed edges are inferred microbial interactions from source to target. The network is constructed based on Lotka-Volterra dynamics (Eq. (1)), which is a conventional way of investigating fluctuations in the populations of wild animals. MetaMIS is the first tool for inferring metagenomic microbial interactions in manner that is automatic and allows for the direct visualization of microbial interaction networks through a user-friendly interface. Figure 1 outlines the rationale of MetaMIS; Fig. 2 depicts the workflow and key features of MetaMIS using screenshots; and Fig. 3 provides a schematic representation of the interrelationships among these features. The detail operation of MetaMIS is introduced in the supplementary (Additional file 1).

The foundation of MetaMIS was the inference of microbial interactions following an abundance-ranking strategy (Fig. 1) that involves ranking OTUs according to their average abundance levels among samples, generating multiple interaction networks and retaining the maximum number of low abundance OTUs in an interaction network (Fig. 1b). This strategy was derived in a straightforward fashion from an empirical rule that dominant microbes are most likely to be observed and analyzed in experimental microbial abundance profiles, and this approach greatly simplifies the complex problem of finding a conserved interaction subnetwork. For each interaction network, there were two possible outcomes (a successful or failed interaction network (Fig. 1a) realized by a generalized form of Lotka-Volterra equation (Eq. (2)). A set of predicted interactions that could successfully regenerate abundance profiles within the prescribed period of time constituted a successful network. Otherwise, failure could be due to inaccurate inference of microbial interactions. The regenerated abundance profiles (successful cases) should be further compared with the original data based on the Bray-Curtis dissimilarity (Eq. (3)). A smaller Bray-Curtis dissimilarity (BCD) would mean that interactions could reproduce microbial abundance similar to the original and were more likely to reveal the underlying interactive relations of a microbial community. These processes are easy to carry out using the user-friendly interface of MetaMIS (Fig. 2).

## Results

### Case study: human intestinal microbiome

In the case study, human fecal microbiomes were collected daily from two healthy subjects, one female, for 6 months, and one male, for 15 months [21], which are publicly available at MG-RAST:4457768.3-4459735.3. The male fecal microbiomes containing more time points were used to demonstrate the functionality of MetaMIS. We constructed 27 interaction networks in total over a span of 420 days, the most compact of which was composed of 14 high abundance families. *Micrococcaceae*, the least abundant among the 14 families, influenced the other 13. According our calculations, *Micrococcaceae* repressed *Oxalobacteraceae*, *Bacteroidaceae*, *Porphyromonadaceae*, *Rikenellaceae*, *Eubacteriaceae*, *Lachnospiraceae*, *Ruminococcaceae*, and *Verrucomicrobiaceae*, but activated *Neisseniaceae* and *Prevotellaceae*. Comparative analysis of the male and female fecal microbiomes using MetaMIS revealed a consensus interaction network.

### Functionality of MetaMIS

Using the greengenes taxonomy, the total number of taxa assigned to the family level was 92 over 317 time points for the male fecal microbiome [21]. Using the default settings of MetaMIS, we detected 14 high abundance families, 22 that were low abundance and not rare, and 56 rare families (Fig. 1a), with a total of 27 interaction networks. Results from an interaction network with the 14 most abundant families are schematized in Fig. 3a–g. In general, the original abundance profiles (Fig. 3a), measured by Eq. (1), seem to present more fluctuation than the predicted ones (Fig. 3b), which were generated by Eq. (2). For each interaction outcome, MetaMIS displayed an interaction network containing the minimum number of strongest interactions to cover all families in this network (Fig. 3D-1): Global view). MetaMIS provides a scrolling bar for users to modify more or less interactions according to interactive strength. In brief, the global interaction network showed *Micrococcaceae* was the least abundant among the 14 families (Table 1), but played the most influential role in the system. A specific view served to display the overall interactive relations of *Micrococcaceae* with the other 13 families (Fig. 3D-2: Specific view). *Micrococcaceae* showed strong negative relations with eight bacterial families, *Oxalobacteraceae*, *Bacteroidaceae*, *Porphyromonadaceae*, *Rikenellaceae*, *Eubacteriaceae*, *Lachnospiraceae*, *Ruminococcaceae*, and *Verrucomicrobiaceae*, and was positively associated with *Neisseriaceae* and *Prevotellaceae* (Fig. 3D-2). In the specific view, weaker interactions with *Micrococcaceae* could be observed with clarity. *Micrococcaceae* acted as a regulator that strongly influenced the other families but was only slightly influenced by them (Fig. 3D-2). It is worth noting that *Micrococcaceae* tended to repress core microbes but to activate none-core taxa (Table 1).

**Table 1:** The male intestinal microbiome was ranked according to the average abundance among 317 time points

MetaMIS ID	Microbial name	Average abundance	Core ratio
ID 1	*Lachnospiraceae*	24.89%	100.00%
ID 2	*Bacteroidaceae*	24.61%	100.00%
ID 3	*Ruminococcaceae*	9.97%	100.00%
ID 4	*Neisseriaceae*	6.00%	2.20%
ID 5	*Unclassified*	4.34%	100.00%
ID 6	*Rikenellaceae*	3.98%	100.00%
ID 7	*Porphyromonadaceae*	3.60%	99.40%
ID 8	*Staphylococcaceae*	3.07%	1.30%
ID 9	*Prevotellaceae*	2.53%	63.40%
ID 10	*Clostridiales Family XI. Incertae Sedis*	2.23%	54.30%
ID 11	*Oxalobacteraceae*	1.40%	95.60%
ID 12	*Eubacteriaceae*	1.30%	99.70%
ID 13	*Verrucomicrobiaceae*	1.21%	76.00%
ID 14	*Micrococcaceae*	1.04%	0.90%
ID 15	*Actinomycetaceae*	0.77%	23.70%
ID 16	*Leptotrichiaceae*	0.67%	0.90%
ID 17	*Enterobacteriaceae*	0.56%	82.00%
ID 18	*Veillonellaceae*	0.54%	55.50%
ID 19	*Fusobacteriaceae*	0.51%	16.40%
ID 20	*Nitrosomonadaceae*	0.50%	59.90%
ID 21	*Dietziaceae*	0.45%	22.40%
ID 22	*Campylobacteraceae*	0.43%	42.90%
ID 23	*Pasteurellaceae*	0.39%	18.30%
ID 24	*Sporolactobacillaceae*	0.36%	0.60%
ID 25	*Chromatiaceae*	0.33%	94.00%
ID 26	*Sphingobacteriaceae*	0.28%	98.70%
ID 27	*Corynebacteriaceae*	0.27%	21.50%
ID 28	*Flavobacteriaceae*	0.26%	1.30%
ID 29	*Streptococcaceae*	0.25%	65.90%
ID 30	*Desulfovibrionaceae*	0.24%	65.00%
ID 31	*Erysipelotrichaceae*	0.23%	98.70%
ID 32	*Dermacoccaceae*	0.19%	3.50%
ID 33	*Blattabacteriaceae*	0.16%	4.70%
ID 34	*Ectothiorhodospiraceae*	0.13%	0.60%
ID 35	*Nocardioidaceae*	0.12%	9.50%
ID 36	*Aerococcaceae*	0.11%	9.10%
ID 37	*Coriobacteriaceae*	0.10%	85.80%
ID 38	*Spirochaetaceae*	0.10%	0.90%
ID 39	*Acidaminococcaceae*	0.09%	76.30%
ID 40	*Intrasporangiaceae*	0.09%	0.60%
ID 41	*Clostridiaceae*	0.09%	98.70%
ID 42	*Acholeplasmataceae*	0.08%	0.30%
ID 43	*Rhodocyclaceae*	0.07%	0.60%
ID 44	*Nocardiaceae*	0.07%	1.90%
ID 45	*Comamonadaceae*	0.06%	5.40%
ID 46	*Bradyrhizobiaceae*	0.06%	4.40%
ID 47	*Enterococcaceae*	0.05%	2.80%
ID 48	*Caulobacteraceae*	0.05%	1.30%
ID 49	*Pasteuriaceae*	0.05%	0.30%
ID 50	*Gemmatimonadaceae*	0.05%	0.90%
ID 51	*Burkholderiaceae*	0.05%	2.50%
ID 52	*Spiroplasmataceae*	0.05%	0.30%

The core ratio represents the percentage frequency of one OTU appeared across time-series samples

Only 52 of 92 OTUs are listed hereThe large core ratio represents that this OTU is present in more time-series samples

Furthermore, three approaches were used to visualize the interactive relations between one microbe and the others (Fig. 3e–g). The most frequent interactive relation for *Micrococcaceae*, i.e., ID14, *was* parasitism or predation (+/−), as shown in Fig. 3e. The interactive strength of each interaction pattern is shown in Fig. 3f. According to the PCA decomposition of the frequency of interaction patterns, ID14 is located in the direction of parasitism or predation (+/−) and amensalism (−/0) (Fig. 3g).

Among 27 successful interaction networks, 18 demonstrated similar predictive power, with BCD (Eq. (3)) ranging from 0.18 to 0.22 (Fig. 3h): 14-OTU, 21-OTU, 37-OTU, …, and 52-OTU. Other than the 14-OTU and 21-OTU interaction networks, 16 rare families participated sequentially in the remaining successful networks, from 37-OTU to 52-OTU. Among these 16 rare families, *Coriobacteriaceae* (core = 85.8%), *Acidaminococcaceae* (core = 76.3%), and *Clostridiaceae* (core = 98.7%) were frequently present at the 317 time points (Table 1) and showed different abundance profiles with others (measured by Pearson correlation among microbial members (0.06, *p* = 0.52)).

### Examining the dependency of interacting pairs

**Fig. 4:**
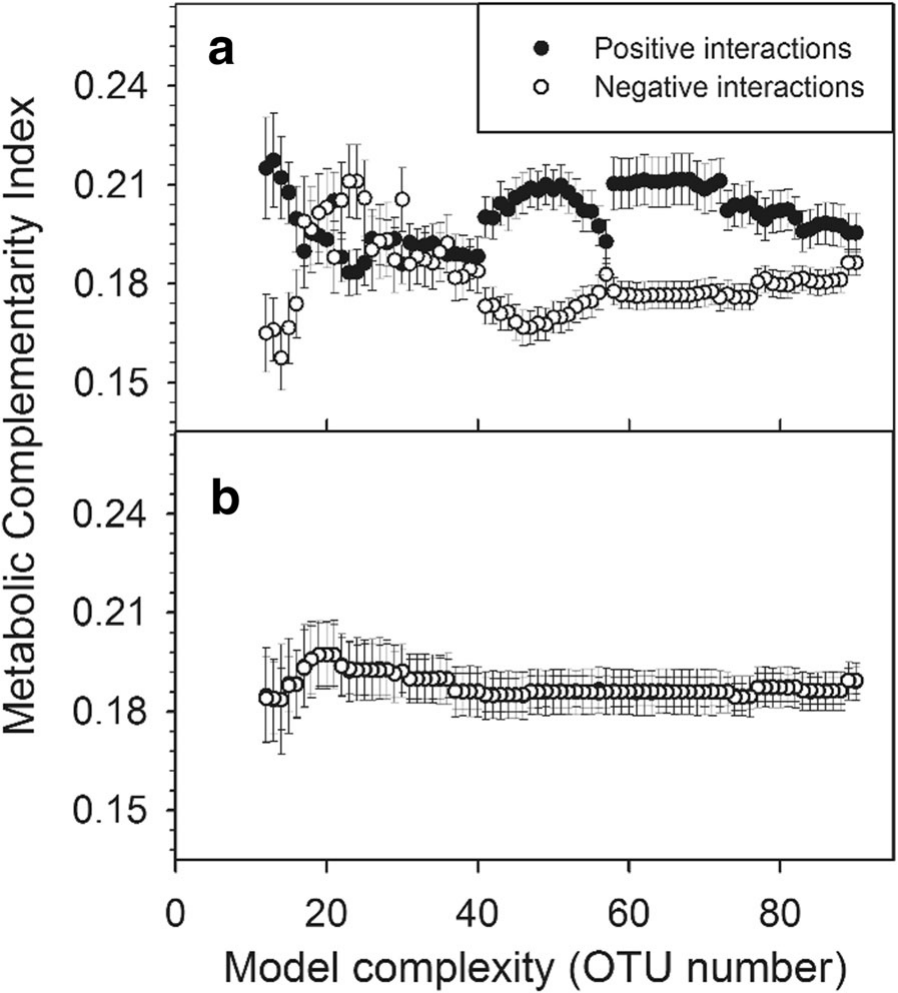
Predicted microbial interactions show biological connections. **a** Positive interactions (*black circles*) were rich in metabolic complementarity. Negative interactions (*white circles*) generally showed lower levels of metabolic complementarity. **b** There were no differences of metabolic complementarity between the two groups in which positive or negative interactions were randomly selected. The error bar represented the standard error of metabolic complementarities for each group

As noted, Lotka-Volterra models have been commonly used to infer animal interactions in ecological studies. For this tool, we applied the Lotka-Volterra model to the investigation of microbial interactions, and further provided a validation calculation by measuring the metabolic complementarity index of the datasets. Metabolic complementarity is an index that measures the trophic relations between two microbes based on a metabolic network [22]. The index may reflect the interdependence of each microbe pair, in which the metabolic waste of one microbe is necessary for the other. We observed that positive interactions within the male intestinal microbiome tended to be associated with a larger metabolic complementary index while negative interactions tended to reach a lower level (Fig. 4a). Alternatively, if the interaction of two microbes is set up randomly, the trophic relations will show no significant difference between two groups (Fig. 4b). Thus, the results using male intestinal microbiomes suggested that the inferred interaction was reasonable.

### Comparative study

MetaMIS is able to organize multiple interaction networks into a consensus interaction network. In this section, we identify consistent microbial interactions among male and female fecal microbiomes via consensus interaction networks. In the analysis of female fecal microbiome, we focused on the influence of rare or low abundance families on the inference of microbial interactions. The female fecal microbiome contained 9 high, 11 non-rare, and 49 rare families. The latter 60 rare or low abundance families were tested to determine their influence on the high abundance 9-OTU interactive network independently. Our results showed that the female intestinal interactive network (BCD = 0.175) was greatly influenced by rare or low abundance families, 7 out of 60 relatively low abundance OTUs showed significant improved effects in generating the interaction profiles (the median of BCD was 0.167, *p* < 0.05, Student’s *t* test).

**Fig. 5:**
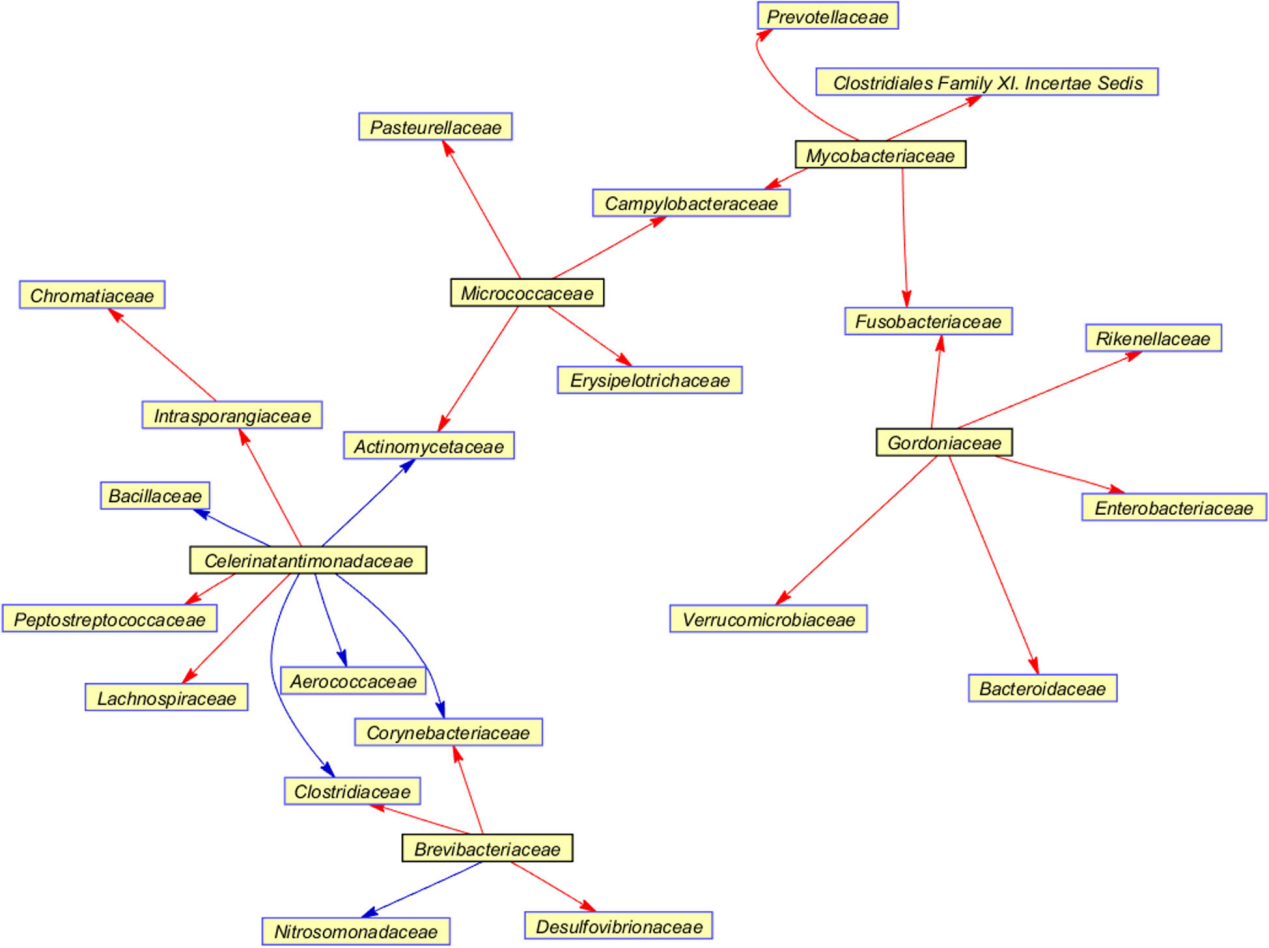
A consensus interaction network of male and female intestinal community. The red (or *blue*) arrow represents the activation (or *repression*)

For each microbiome (male and female), a consensus interaction network was organized from the comparison of all interaction networks using one sample z-test for proportions, instead of measuring the change of interaction strengths. The female microbiome, containing 69 families over 124 time-series points, in which 63 were overlapped with the male microbiome, generated 1,128 confident positive interactions and 937 negative interactions. The male microbiome produced more interactions in its consensus network, for a total of 1,618 positive and 2,643 negative interactions. With regard to the absolute interactive strength, 26 stronger interactions among 26 families were coherent between the male and female microbiomes (Fig. 5). The relative abundance or core ratio of 26 families is shown in Table 2. Acting as transmitters, the rare families *Celerinatantimonadaceae*, *Micrococcaceae, Brevibacteriaceae, Gordoniaceae*, and *Mycobacteriaceae* played key roles to influence others. *Celerinatantimonadaceae* repressed four rare or low abundance non-core families, *Bacillaceae*, *Actinomycetaceae*, *Aerococcaceae*, and *Corynebacteriaceae*, and one rare core families, *Clostridiaceae*. However, *Micrococcaceae* and *Brevibacteriaceae* tended to activate low level non-core families. *Gordoniaceae* had strong positive association with high abundant core families, *Verrucomicrobiaceae, Bacteroidaceae, Enterobacteriaceae*, and *Rikenellaceae. Mycobacteriaceae* colonized in male intestinal tracts activated two highly abundant non-core families, *Prevotellaceae* and *Clostridiales Family XI. Incertae Sedis*. The community of these highly abundant families, acting as receptors, seemed to be greatly influenced by rare or low abundance microbes. Furthermore, *Micrococcaceae* was also identified as an influential bacterial family, not only in the male 14-OTU interaction network, but also in this consensus interaction network, reflecting its common role in the male and female biomes.

**Table 2:** OTUs showing consistent microbial interactions between male and female intestines and their taxonomic abundance and core levels

	Female		Male	
	ABUN	CORE	ABUN	CORE
*Lachnospiraceae*	34.61%	100.00%	24.89%	100.00%
*Bacteroidaceae*	31.04%	100.00%	24.61%	100.00%
*Verrucomicrobiaceae*	8.90%	96.77%	1.21%	76.03%
*Enterobacteriaceae*	1.44%	78.23%	0.56%	82.02%
*Nitrosomonadaceae*	1.04%	72.58%	0.50%	59.94%
*Corynebacteriaceae*	0.49%	21.77%	0.27%	21.45%
*Erysipelotrichaceae*	0.40%	100.00%	0.23%	98.74%
*Prevotellaceae*	0.37%	83.87%	2.53%	63.41%
*Desulfovibrionaceae*	0.27%	91.94%	0.24%	64.98%
*Chromatiaceae*	0.26%	54.03%	0.33%	94.01%
*Fusobacteriaceae*	0.10%	34.68%	0.50%	16.40%
*Clostridiales Family XI. Incertae Sedis*	0.09%	59.68%	2.23%	54.26%
*Rikenellaceae*	0.06%	87.90%	3.98%	100.00%
*Pasteurellaceae*	0.05%	38.71%	0.39%	18.30%
*Peptostreptococcaceae*	0.04%	80.65%	0.03%	65.30%
*Micrococcaceae*	0.04%	3.23%	1.04%	0.95%
*Mycobacteriaceae*	0.04%	4.03%	0.02%	1.89%
*Clostridiaceae*	0.03%	90.32%	0.09%	98.74%
*Campylobacteraceae*	0.03%	30.65%	0.43%	42.90%
*Aerococcaceae*	0.02%	21.77%	0.11%	9.15%
*Bacillaceae*	0.02%	49.19%	0.02%	28.08%
*Actinomycetaceae*	0.02%	29.03%	0.77%	23.66%
*Brevibacteriaceae*	0.01%	4.84%	0.03%	0.32%
*Intrasporangiaceae*	0.01%	1.61%	0.09%	0.63%
*Gordoniaceae*	0.01%	1.61%	0.03%	0.63%
*Celerinatantimonadaceae*	0.01%	0.81%	0.03%	0.63%

## Discussions and conclusions

The Lotka-Volterra equations, which are canonical in mathematical ecology, provide variable ways to illustrate the importance of nonlinear dynamics [23]. Recently Lotka-Volterra models have been applied in the field of metagenomics to investigate microbial interactions because of their usefulness in reverse-engineering multispecies ecosystems [17, 18]. In this context, these models serve to simulate multi-species microbial communities with known interaction relations [10, 11, 15] that can be adjusted for systematic stability analysis [15]. Recent work, including studies of yeast-bacterium interactions on the surface of cheese [18] and microbial interactions in murine intestinal communities [17], have demonstrated that Lotka-Volterra models can be used to reverse-engineer the interactive behaviors of an ecosystem, even in response to such external perturbations as antibiotic intervention. These studies are important for understanding the application of Lotka-Volterra models to the comprehensive inference of dynamic biological systems in the effort to decipher the interrelationships between species.

In this paper, we have presented a user-friendly, stand-alone GUI tool, MetaMIS, that is designed to provide rapid and accurate predictions of microbial interactions that can help to reveal temporal changes in microbial communities. The integrated diagrammatic presentation can aid in revealing mechanically interactive links between microbes. We offered as examples three interaction networks inferred from a human male, female, and a mixed-gender fecal microbiome. Those inferred relationships receive some support in the literature. For example, some strains of *Micrococcaceae* have been shown to possess considerable antibacterial activity [24] and antibiotic-resistance ability that counters the inhibitory effect of *Lactobacillus, Lact. sake CL35* [25]. Furthermore, we found that *Micrococcaceae* consistently activated two microbes, *Neisseriaceae* and *Prevotellaceae*, which is consistent with the studies showing that the use of antibiotic agents significantly increases the incidence of members of the *Prevotellaceae* family in the mucosal-associated microbiome [26]. The antimicrobial effect of *Micrococcaceae* [24] and *Neisseriaceae* [27] might therefore balance those dominant microorganisms and thereby help to maintain innate homeostasis and to achieve a more diverse intestinal ecosystem. Overall, these reported microbial functions and characteristics were consistent with the microbial interactions that we inferred.

In the case of consensus network, *Mycobacteriaceae*, which is defined as a rare family in the male microbiome and a non-core family in the female microbiome, and is associated with tuberculosis [28, 29], also exhibited a similar interaction pattern in both genders. On the other hand, several studies have noted that sex hormones and microbes together trigger a gender bias in such autoimmune diseases as type 1 diabetes (T1D) [30] and systematic lupus erythematosus (SLE) [31]. As suggested, the distribution of *Enterobacteriaceae* and *Peptostreptococcaceae* correlated strongly with the concentration of androgen as conditions in which male nonobese diabetic (NOD) mice experienced a lower risk of T1D [30]. However, our data suggest that the role of *Enterobacteriaceae* and *Peptostreptococcaceae* in the male and female samples could be the same considering the interaction patterns with the other microbes. Furthermore, we suggest that, when analyzing metagenomic abundance profiles, considerable care is required in determining the cutoff for low abundance or rare OTUs, informative interactions from low level members may be lost.

## Conclusion

In sum, here we have presented an easy-to-follow workflow designed to infer microbial interactions using Lotka-Volterra models for 16S-rRNA microbial abundance profiles. MetaMIS allows researchers to analyze interactive relations conveniently and to visualize network topology directly through an intuitive graphic user interface. The abundance-ranking strategy of MetaMIS produces a variety of interaction networks and allows maximum information to be gathered regarding low-abundance members of the microbial community. Among different interaction networks, users can trace changes in interactive relations or utilize a consensus network that contains a set of OTUs with qualified interactions in order to identify key microbes. The publicly available MetaMIS is expected to undergo continuous development; future plans include: organizing interaction networks across different dataset, establishing topological analyses to extract key OTUs based on their topological nature, plugging in a functional annotated package for microorganisms, and, in the longer term, developing a pathway dependent interaction cascade. We view the current version of MetaMIS as a first step toward facilitating the interpretation of metagenomic studies in the context of the rapidly expanding knowledge of microbial genomes and the growing databases that store that knowledge.

## Methods

### Implementation

MetaMIS was performed as an off-line GUI coded by a commercial software package (MATLAB R2015b, The MathWorks, Inc., Natick, Massachusetts, United States). It runs properly on Mac and Windows (64-bit) platforms. Before the execution of MetaMIS, the Matlab runtime should be installed, which is a simple one-click process.

### Data preprocessing

Before they use MetaMIS, we recommended that users perform two kinds of data preprocessing for a metagenomic microbial abundance profile. First, 16S rRNA amplicon microbial profiles should be corrected based on 16S rRNA gene copy number (GCN) information, since GCN bias may compromise the accuracy of microbial abundance profiles and significantly influence biological interpretations [32]. Second, microbial abundance profiles should be normalized by transformation to relative abundance, which is done by dividing the minimum number of total reads for all samples, and finally deleting OTUs without abundance values for all samples. The aim of this process is to ascertain which low abundance OTUs are present.

### The classification of OTUs according to population size

According to the average abundance across samples in which the zero count was not included in the average calculation, microbial OTUs may be categorized into three groups as follows. The high abundance group is characterized by OTUs with average abundance greater than 1%. Rare species are characterized by an average abundance lower than 0.1%. The remaining organisms are assigned to the low abundance, non-rare group.

### The inference of microbial interactions

In a metagenomic microbial abundance profile, there are *i* =1,…,*L* microbes or taxonomic labels, i.e. OTUs, and *k* =1,…,*T* time points. Time-series samples with total reads smaller than 5,000 are automatically deleted in MetaMIS. Next, a discrete-time Lotka-Volterra model (Eq. (1)) [33] coupled with a partial least square regression (PLSR) is used to infer microbial interactions, from which the number of PLS components containing the minimum estimated mean-squared error is determined. PLSR is a powerful method for handling a highly correlated time series data structure [34].

1 $$ \frac{ \ln \left({x}_i\left({t}_{k+1}\right)\right)- \ln \left({x}_i\left({t}_k\right)\right)}{t_{k+1}-{t}_k}={r}_i+{\sum}_{j=1}^L{M}_{ij}{x}_j\left({t}_k\right) $$

where *x*_*i*_(*t*_*k*_) represents microbial abundances for any OTU *i* at the time *t*_*k*_, *r*_*i*_ is the growth rate of OTU *i*, and *M*_*ij*_ characterizes the interactive effect of OTU *j* on *i*. In general, *M*_*ij*_ > 0 means that OTU *j* has an activated ability to OTU *i*, while *M*_*ij*_ < 0 means that the repressive effect of OTU *j* on *i*, and *M*_*ij*_ = 0 shows no interaction between OTU *i* and *j*. Notice that MetaMIS chooses the components as predictors using above method, the result may effect the estimated interaction strengths and signs.

### The criteria for a successful interaction network

After microbial interactions have been estimated, they can be placed into a generalized Lotka-Volterra model (Eq. (2)) [33] in order to evaluate the possibility of regenerating microbial profiles over time *T*. The initial condition can be any time-series sample. The default setting is the first one.

2 $$ \frac{d}{dt}{x}_i\left({t}_k\right)={r}_i{x}_i\left({t}_k\right)+{x}_i\left({t}_k\right){\sum}_{j=1}^L{M}_{ij}{x}_j\left({t}_k\right) $$

A set of microbial interactions is considered to constitute a successful interaction network when microbial abundance profiles can be successfully regenerated using estimated microbial interactions over time *T*. If regenerated abundances meet the divergence before the end of the threshold time *T*, the corresponding microbial interactions represent the failure to form an interaction network.

For each successful interaction network, the concordance between the predicted abundance profiles and the original ones was measured by Bray-Curtis dissimilarity (Eq. (3)) [35].

3 $$ BCD\left({x}_{i{t}_k},{x}_{i{t}_k}^{*}\right)=\frac{{\displaystyle {\sum}_{i=1}^L}\left|{x}_{i{t}_k}-{x}_{i{t}_k}^{*}\right|}{{\displaystyle {\sum}_{i=1}^L}\left({x}_{i{t}_k}+{x}_{i{t}_k}^{*}\right)} $$

where ${x}_{i{t}_k}^{*}$ is the estimated microbial abundance of OTU *i* at the *t*_*k*_. This index ranges from 0 and 1. The larger the value, the more dissimilar are the two abundance profiles, and vice versa.

### The filtering thresholds for interaction networks

A microbial community with N OTUs can generate N-N_HA_ + 1 interaction networks by the default settings of MetaMIS, where N_HA_ ≥3 represents the number of high abundance OTUs. The initial N-dimensional network contained N(N-1) interactions from weakest to strongest in the entire community. Then, an OTU with lowest abundance value was discarded and the remaining N-1 OTUs produced (N-1) (N-2) interactions. The strategy of leaving the lowest one out was performed until there were only N_HA_ high abundance OTUs in an interaction network.

For these N- N_HA_ + 1 interaction outcomes, one sample z-test for proportions was used to measure the concordance of predicted interactive relations among networks. For an interaction pair, *M*_*ij*_, there were *n*_*ij*_^+^ and *n*_*ij*_^−^ interaction networks producing positive and negative outcomes when the interactive direction was fixed. When the ratio of *n*_*ij*_^+^ to the summation of *n*_*ij*_^+^ and *n*_*ij*_^−^ was statistically significantly greater than the user-defined threshold for this study, i.e., 90%, we were able to conclude that this interaction relation was concordant among networks and directed positively, and vice versa.

### The evaluation of inferred microbial interactions

Microbial interactions predicted by MetaMIS were evaluated using a metabolic complementarity index [22]. The metabolic complementarity ranges from 0 to 1 as a measure of the trophic dependence between two microbes; thus an index of 1 means that all of the nutrients required by one microbe can be synthesized by another microbe from metabolic precursors. In a recent study [22], 23,562 trophic interactions between pairs of microbial species based on genome-scale metabolic network models were transformed to 19,182 microbial interactions on the family level. For each transformation, indices from the common family were averaged.

Mapping the trophic information into predicted interactions from MetaMIS was done in order to explore the underlying biological connections. For each interaction network, network size was a key component of the mapping rate, 9.7 to 62.9% for the male gut and 15.9 to 62.2% for the female gut. A larger network size usually correlated with a lower mapping rate.

## Additional file

Additional file 1: User guide of MetsMIS. (PDF 4192 kb)

## Abbreviations

BCD: Bray-Curtis dissimilarity
GUI: Graphic user interface
LIMITS algorithm: The Learning Interactions from MIcrobial Time Series algorithm
MetaMIS: **Meta**genomic **M**icrobial **I**nteracticon **S**imulator
NOD mice: Nonobese diabetic mice
OTUs: Operational taxonomic units
RMN algorithm: The Rule-based Microbial Network algorithm
rRNA: ribosomal RNA
SLE: Systematic lupus erythematosus
SparCC: Sparse Correlations for Compositional data
SPIEC-EASI algorithm: The SParse InversE Covariance estimation for Ecological ASsociation Inference
T1D: Type 1 diabetes
