# Supplementary material for: MetaMIS: a metagenomic microbial interaction simulator based on microbial community profiles
Source: BMC Bioinformatics. 2016 Nov 25;17:488. doi: 10.1186/s12859-016-1359-0 (PMC5124289; doi:10.1186/s12859-016-1359-0)
Supplement: Additional file 1: — User guide of MetsMIS. (PDF 4192 kb) [file 12859_2016_1359_MOESM1_ESM.pdf]

Developers

Grace Tzun-Wen Shaw (tzunwen@gmail.com)

Yueh-Yang Pao (greanozone@gmail.com)

Daryi Wang (dywang@gate.sinica.edu.tw)

Dr. Daryi Wang’s Laboratory

Biodiversity Research Center, Academia Sinica

Contents

1. What MetaMIS can do? 2
2. Support platform....3
3. Installation 3
4. MetaMIS Input Format 4
    4.1 Data format 4
    4.2 Test data: 5
5. Run MetaMIS 6
    5.1 Graphical user interface of MetaMIS: 6
    5.2 Pipeline for data flow and parameter selection: 6
    5.3 Example of Test data: 8
6. MetaMIS Results 10
    6.1 Five tab panels for MetaMIS outputs 10
    6.2 Export File 19

Bugs or problems 25

References....26

1. What MetaMIS can do?

MetaMIS (**Meta**genomic **M**icrobial **I**nteraction **S**imulator) is a Lotka-Volterra model based tool to infer microbial interactions based on microbial community. The functionalities of MetaMIS are listed as follows.

1. Automatically infer microbial interactions from 16S rRNA abundance profiles based on abundance-ranking strategy.
2. Visualize the predicted abundance profiles
3. Visualize the interaction networks
4. MetaMIS systematically examines interaction patterns, such as mutualism (+/+), competition (-/-), parasitism or predation (+/-), commensalism (+/0), amensalism (-/0), and no effect (0/0).
5. Principal component analysis (PCA) is embedded to refine the biological role inside microbes.
6. Provide a consensus network from multiple interaction netwroks.
7. The interaction tables generated by MetaMIS can be exported as Gephi or Cytoscape format for advanced topological analysis
8. MetaMIS is publicly free and accessible at https://sourceforge.net/projects/metamis/ without login requirement.

2. Support platform

MetaMIS was developed and maintained on Matlab (MATLAB R2015b, The MathWorks, Inc., Natick, Massachusetts, United States). Currently, MetaMIS supports Windows (64 bits) and Mac platform.

3. Installation

https://sourceforge.net/projects/metamis/

Please install Matlab runtime before running MetaMIS.

(If your desktop has installed the Matlab software already, please skip the runtime installation.)

GUI version:

Windows (64bits)

1. Matlab runtime program: MatlabRuntim_Installer.exe
2. Main program: MetaMIS.exe

Mac

1. Matlab runtime program: MatlabRuntim_Installer.app
2. Main program: MetaMIS.app

4. MetaMIS Input Format

4.1 Data format

MetaMIS accepts a temporal microbial abundance profile as input in which microbial communities should be obtained in a series of temporal samples. A dataset with long time lapses between consecutive data points may have potential problem, the sparse data may cause false results. To simplify the process of data input, we have defined the input file format as follows. First, a temporal microbial abundance profile needs to be processed in advance according to a user-defined taxonomic level. The data must be in a tab separated text format, described as follows (Fig. 1).

1. OTU identifiers are listed in the first column. The first cell can be empty. Duplicated OTU identifiers are not allowed.
2. The first row is reserved for time-series sample identifiers that must be numeric; and the remaining spaces should be filled with the microbial abundance values, a numeric matrix.
3. Reads or relative abundance tables are both acceptable.

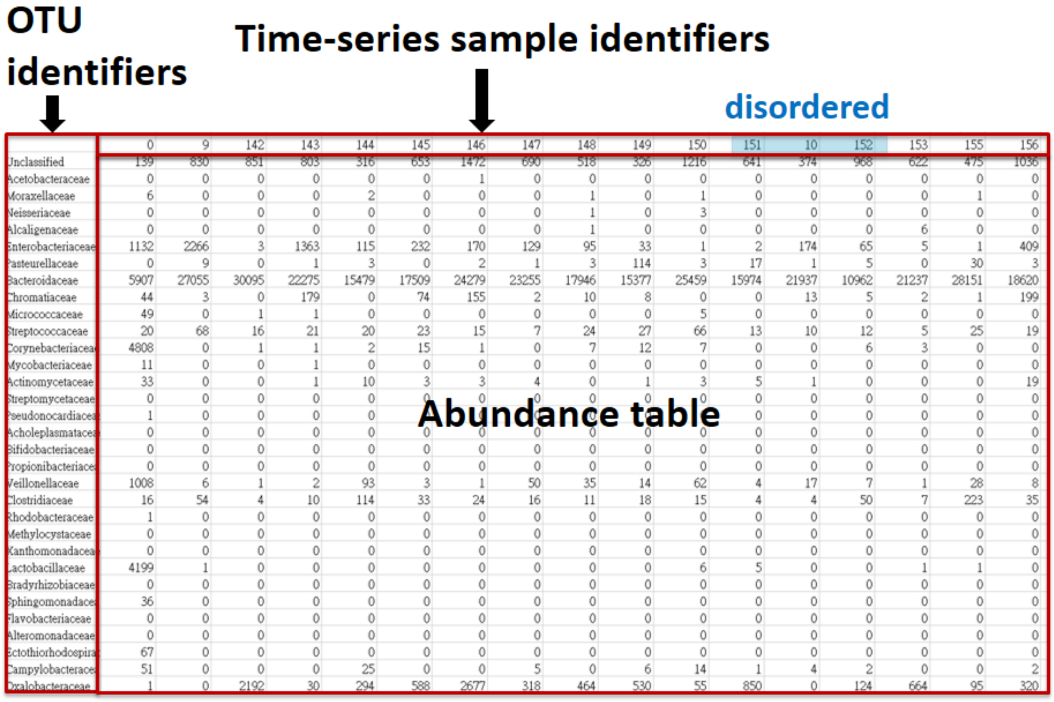

	0	9	142	143	144	145	146	147	148	149	150	151	10	152	153	155	156
Unclassified	139	830	851	803	316	653	1472	690	518	326	1216	641	374	968	622	475	1036
Acetobacteraceae	0	0	0	0	0	0	1	0	0	0	0	0	0	0	0	0	0
Moraxellaceae	6	0	0	0	2	0	0	0	1	0	1	0	0	0	0	1	0
Neisseriaceae	0	0	0	0	0	0	0	0	1	0	3	0	0	0	0	0	0
Alcaligenaceae	0	0	0	0	0	0	0	0	1	0	0	0	0	0	6	0	0
Enterobacteriaceae	1132	2266	3	1363	115	232	170	129	95	33	1	2	174	65	5	1	409
Pasteurellaceae	0	9	0	1	3	0	2	1	3	114	3	17	1	5	0	30	3
Bacteroidaceae	5907	27055	30095	22275	15479	17509	24279	23255	17946	15377	25459	15974	21937	10962	21237	28151	18620
Chromatiaceae	44	3	0	179	0	74	155	2	10	8	0	0	13	5	2	1	199
Micrococcaceae	49	0	1	1	0	0	0	0	0	0	5	0	0	0	0	0	0
Streptococcaceae	20	68	16	21	20	23	15	7	24	27	66	13	10	12	5	25	19
Corynebacteriacea	4808	0	1	1	2	15	1	0	7	12	7	0	0	6	3	0	0
Mycobacteriaceae	11	0	0	1	0	0	0	0	0	0	0	0	0	0	0	0	0
Actinomycetaceae	33	0	0	1	10	3	3	4	0	1	3	5	1	0	0	0	19
Streptomycetaceae	0	0	0	0	0	0	0	0	0	0	0	0	0	0	0	0	0
Pseudonocardiacea	1	0	0	0	0	[illegible]	[illegible]	[illegible]	[illegible]	[illegible]	[illegible]	0	0	0	0	0	0
Acholeplasmatacea	0	0	0	0	0	0	0	0	0	0	0	0	0	0	0	0	0
Bifidobacteriaceae	0	0	0	0	0	0	0	0	0	0	0	0	0	0	0	0	0
Propionibacteriace	0	0	0	0	0	0	0	0	0	0	0	0	0	0	0	0	0
Veillonellaceae	1008	6	1	2	93	3	1	50	35	14	62	4	17	7	1	28	8
Clostridiaceae	16	54	4	10	114	33	24	16	11	18	15	4	4	50	7	223	35
Rhodobacteraceae	1	0	0	0	0	0	0	0	0	0	0	0	0	0	0	0	0
Methylocystaceae	0	0	0	0	0	0	0	0	0	0	0	0	0	0	0	0	0
Xanthomonadacea	0	0	0	0	0	0	0	0	0	0	0	0	0	0	0	0	0
Lactobacillaceae	4199	1	0	0	0	0	0	0	0	0	6	5	0	0	1	1	0
Bradyrhizobiaceae	0	0	0	0	0	0	0	0	0	0	0	0	0	0	0	0	0
Sphingomonadace	36	0	0	0	0	0	0	0	0	0	0	0	0	0	0	0	0
Flavobacteriaceae	0	0	0	0	0	0	0	0	0	0	0	0	0	0	0	0	0
Alteromonadaceae	0	0	0	0	0	0	0	0	0	0	0	0	0	0	0	0	0
Ectothiorhodospira	67	0	0	0	0	0	0	0	0	0	0	0	0	0	0	0	0
Campylobacterace	51	0	0	0	25	0	0	5	0	6	14	1	4	2	0	0	2
Oxalobacteraceae	1	0	2192	30	294	588	2677	318	464	530	55	850	0	124	664	95	320

Figure 1 Data input format.

4.2 Test data:

1. Data description:

   A gut meategnomic study containing temporal microbiota from a male and a female was used [1]. Using greengenes taxonomy, the number of total taxa assigned at family level were 92 and 69 for male and female microbiomes. The two datasets were further checked by gene copy number correction program [2].

2. Input file:

   (A) M_gut.txt: 92 families over 317 time points

   (B) F_gut.txt: 69 families over 124 time points

3. Output files:

   (A) M_gut.mat

   (B) F_gut.mat

   (C) F_gut_EDGE_9.txt

   (D) F_gut_NODE_9.txt

5. Run MetaMIS

5.1 Graphical user interface of MetaMIS:

There are two main blocks in MetaMIS (Fig. 2).

1. Data loading and preprocess blocks.
2. Visualization of interaction network and analysis.

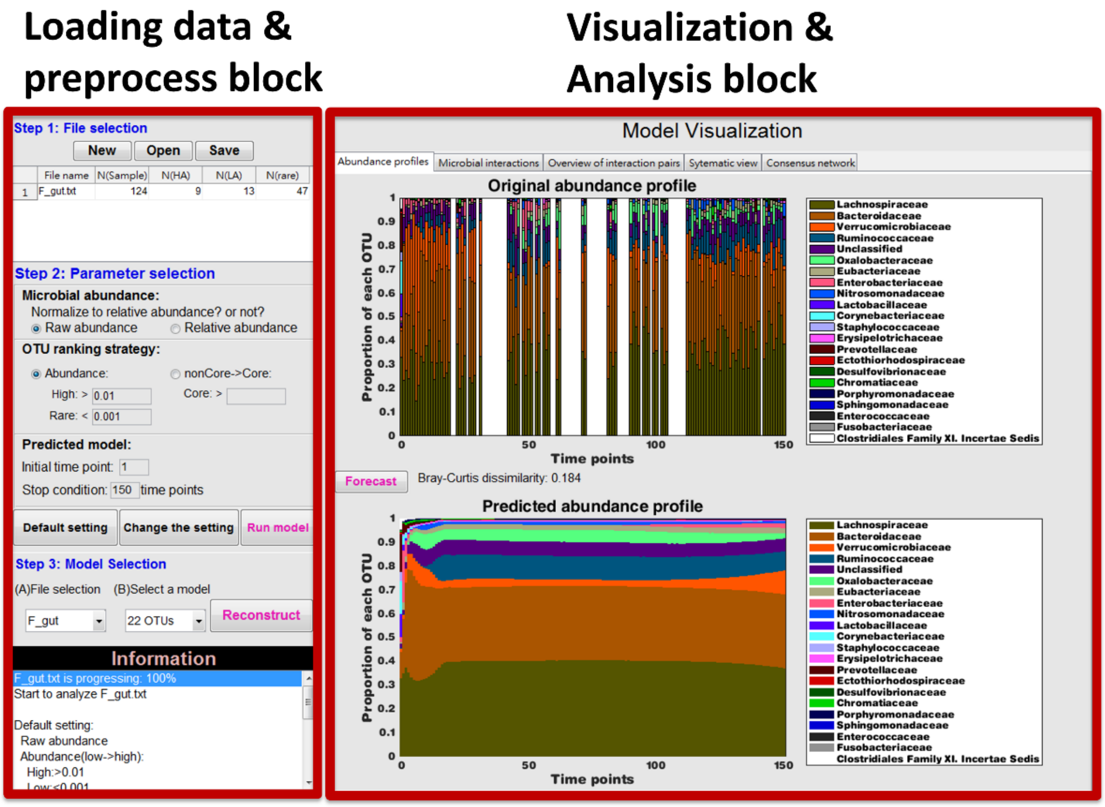

Figure 2 Graphical user interface of MetaMIS.

5.2 Pipeline for data flow and parameter selection:

A typical analysis workflow may contain three steps: uploading of formulated data file(s) (Step 1: File selection), specification of the parameters (Step 2: Parameter selection), and performing the model calculations. Input parameters are optional for users or can be set by default.

5.2.1 Step 1: File selection

Select「New」 to create a new project, e.g. F_gut.txt (Fig. 3).

Select「Open」 to open a processed dataset, e.g. F_gut.mat.

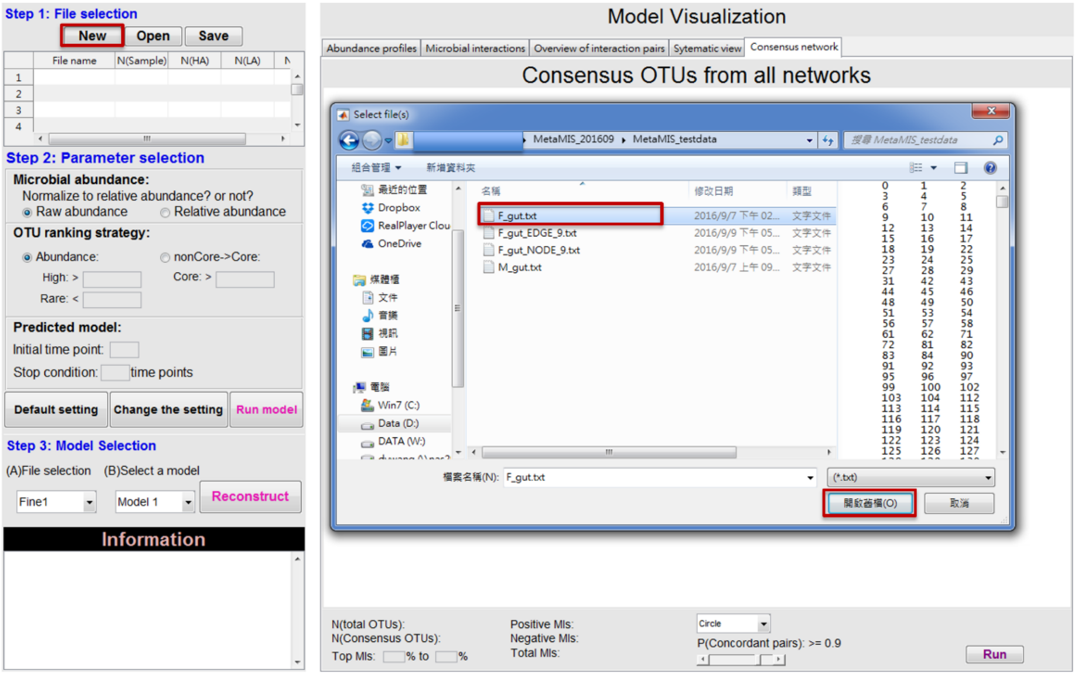

Figure 3 The exemplification of creating a new project.

5.2.2 Step 2: Parameter selection

⊙Microbial abundance:

「Raw abundance」：Time-series samples retain the original input reads.

「Relative abundance」：All samples are adjusted to the common total reads.

⊙OTU ranking strategy:

「Abundance」：OTUs are ranked based on the ratio of average abundance across samples to the total abundance. The default setting of the ratio is >1% for high abundance, <0.1% for rare, and the remaining for low abundance non-rare OTUs. However, the minimum number of

high abundance OTUs is set to be three in MetaMIS owing to the model calculations. If the requirement is not fulfilled, the upper bound of the ratio will be automatically changed to satisfy the mentioned condition.

「nonCore->Core」: Core OTUs are OTUs that consistently present across time-series samples. The default setting of core OTUs is 90%, meaning that those OTUs have detectable reads among over 90% of time-series samples.

⊙Predicted model:

Using generalized Lotka-Volterra equations [3], the inferred microbial interactions can be sued to regenerate abundance profile. In this regeneration process, there are two parameters to be set.

「Initial time point」: According to the input data structure, users can arbitrarily determine any time-series sample as the initial state. Different initial condition may conduct different predicted abundance profiles. The default is the first time-series sample.

「Stop condition」: Whether the regenerated profile can be produced till the end of the stop time point is defined as an indicator of success or failure of inferred interactions. The default setting is 80% of time-series sample size.

5.2.3 Step 3: model calculations

「Run model」: Press this button to perform model calculations based on Lotka-Volterra equations to get abundance-ranking interaction networks.

5.3 Example of Test data:

After F_gut.txt is imported into MetaMIS (Fig. 3), MetaMIS immediately generates the basic data description: there are 124 time-series samples, 9 high abundance, 13 low-abundance non-rare, and 47 rare OTUs in this dataset (Fig. 4(A)).

Case 1:

While pressing the button of 「Default setting」, parameters are automatically determined as follows (Fig. 4(A)).

- 「Raw abundance」 to retain the original data format.
- 「Abundance」:
  - 「High: > 0.01」 to select high abundance OTUs.
  - 「Rare: < 0.001」 to select rare OTUs.
- 「Initial time point」 is set to be the first time point.
- 「Stop condition」 is set to be 80% of terminal time points.

Case 2:

Pressing the button of 「Change the setting」, users can easily modify the parameters. Then, MetaMIS will update the data structure (Fig. 4(B)). The threshold of high abundance OTUs was set to 1 in this test data, and the other parameters adopted default values. MetaMIS will automatically adjust the high abundance threshold to make sure that there are at least three high abundance OTUs in an interaction network.

Once the parameter setting is completed, please press the button 「Run model」 to manipulate the Lotka-Volterra equations for inferring microbial interactions.

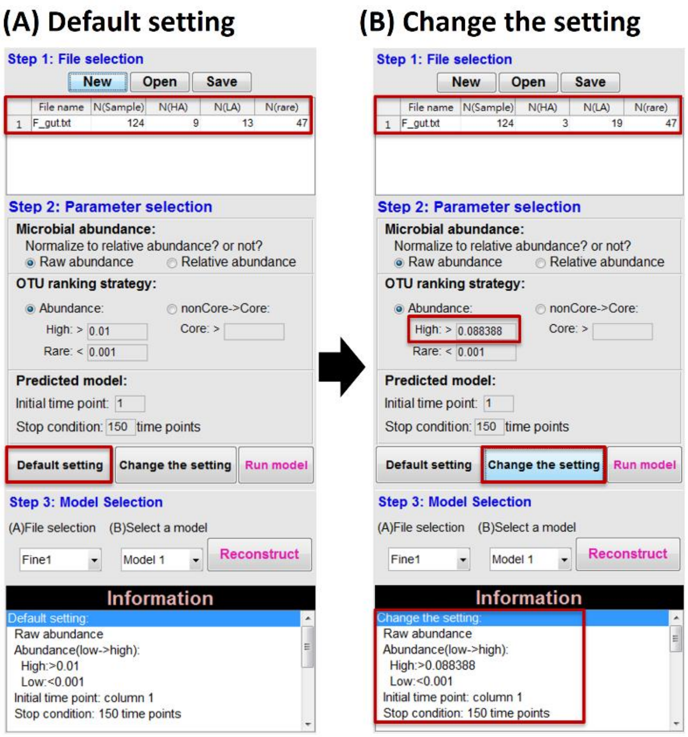

Figure 4 Parameter selection. (A) Press the button of 「Default setting」. (B) Press the button of 「Change the setting」.

6. MetaMIS Results

6.1 Five tab panels for MetaMIS outputs

After the implement of MetaMIS on the female gut communities [1], we got totally 17 interaction networks. As shown in Figure 5, the output page contains five main tab panels to display the selected interaction network. The rightest two panels convey the systematic information from all produced interaction networks. More detailed information is described below from Fig. 5-16.

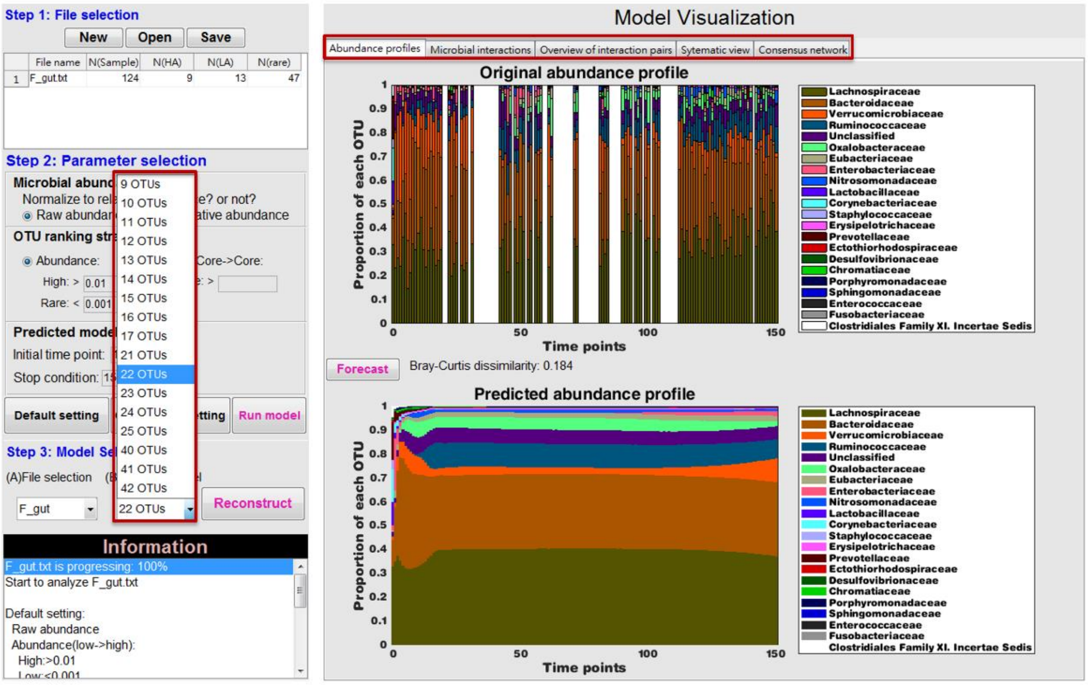

Figure 5 MetaMIS platform showing the original and predicted abundance profiles in the tab panel of "Abundance profiles". The x-axis is time point and the y-axis is the proportion of each OTU at a time-series sample.

6.1.1 Panel of "Abundance profiles" (Fig. 5)

Original abundance profile directly derived from the input dataset contained 124 time points, which could be discontinuous.

Predicted abundance profile is the data generated (abundance profiles) from the

selected interaction network. If users press the button 「Forecast」, MetaMIS produces another predicted one containing all continuous time points. The stop time point is an option for user to select a preferred terminal time point.

Bray-Curtis dissimilarity, ranged from 0 to 1, measures the difference between the original and predicted abundance profiles. The lower the Bray-Curtis (BC) score, the more similar the two abundance profiles. In the test case, interaction network with 22 OTUs conveyed a BC score 0.184, reflecting the reliability of the generated dataset.

6.1.2 Panel of "Microbial interactions"

Inferred microbial interactions are displayed in two manners. The tabular structure shows the quantitative interactive relationships. The network topology is to visualize the microbial interactions (Fig. 6). Furthermore, the interaction strengths can be optionally chosen for users. There are three strength types (Fig .7) in MetaMIS. If different interaction strength type is chosen, the interaction network will be generated based on corresponding strengths.

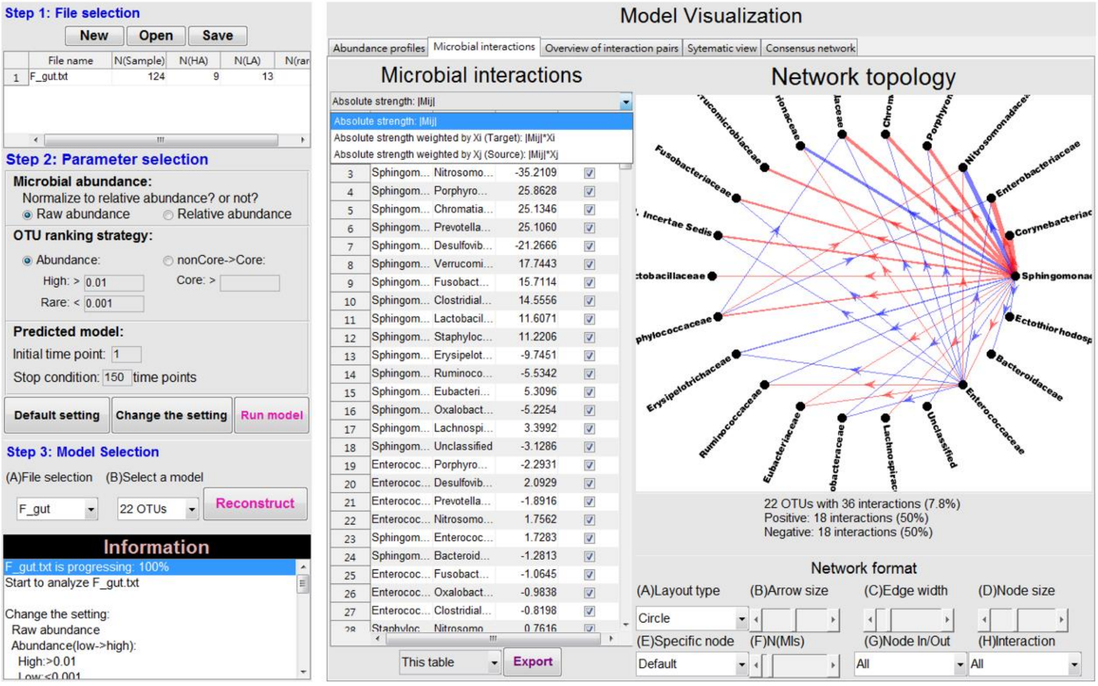

Figure 6 Panel of "Microbial interactions" displays inferred interactions by tabular and network topological view. Interactions were ranked according to the interaction strengths. The blue (or red) line means negative (or positive) interaction between microbes.

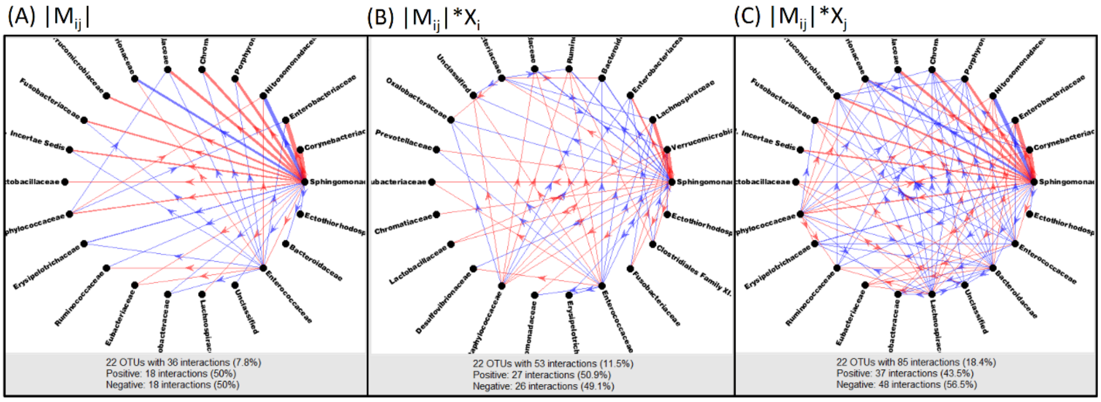

Figure 7 Different types of interaction strengths. (A)$|M_{ij}|$ is absolute interaction strength which can be weighted by target (B) or source abundance (C).

- ◘$|M_{ij}|$: Considering positive or negative strength had similar impacts on interaction networks, interaction strengths are presented by the absolute values.
- ◘$|M_{ij}|*X_i$: Considering abundance values may influence the interaction strengths, all absolute interaction strengths are multiplied by the abundance of target OTUs.
- ◘$|M_{ij}|*X_j$: All absolute interaction strengths are multiplied by abundance of source OTUs.

In the case of test data, there were totally 36 interactions among 22 OTUs. The minimum numbers of interactions covering 8 OTUs are presented in the interactive network (Fig. 6). There are eight optional items in MetaMIS for users to modify the final appearance of the microbial interactive network.

(A) 「Layout type」:

To keep track of visual-spatial information, we provided four kinds of network topological layouts, such as circle, force, layered, and subspace for optional visualization (Fig. 8).

(B) 「Arrow size」:

The arrow means the direction of influence from a source OTU to a target one. Users can fine-tune the arrow size to optimize the network visualization.

(C) 「Edge width」:

The edge width is proportional to the microbial interactive strength. The larger

edge width conveys a stronger microbial interactive strength. Users can change the edge width to highlight or ignore the interactions manually.

(D) 「Node size」：

Each node denotes an OTU. Dragging the scroll bar can change the node size.

(E) 「Specific node」：

“Specific node” allowed users to partially observe the connective behaviors of a specific OTU (Fig. 9). The default setting is to show the interactive network of all nodes in a global view.

(F) 「N(MIs)」：

How many interaction pairs are selected to display in the network (Fig. 9).

(G) 「Node In/Out」：

Select the option of “Node In” (or “Node Out”), and microbial interactions will converge to (or diverge from) a specific OTU (Fig. 10).

(H) 「Interaction」：

If the positive or negative interactions are concerned, this is an optional item to change the status (Fig. 11).

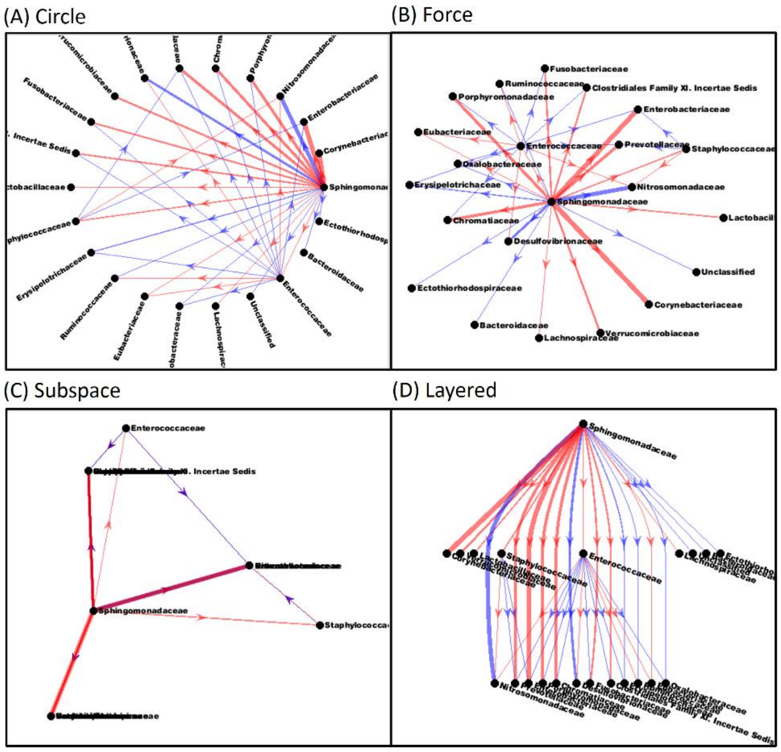

Figure 8 Four kinds of different layout types for network topology.

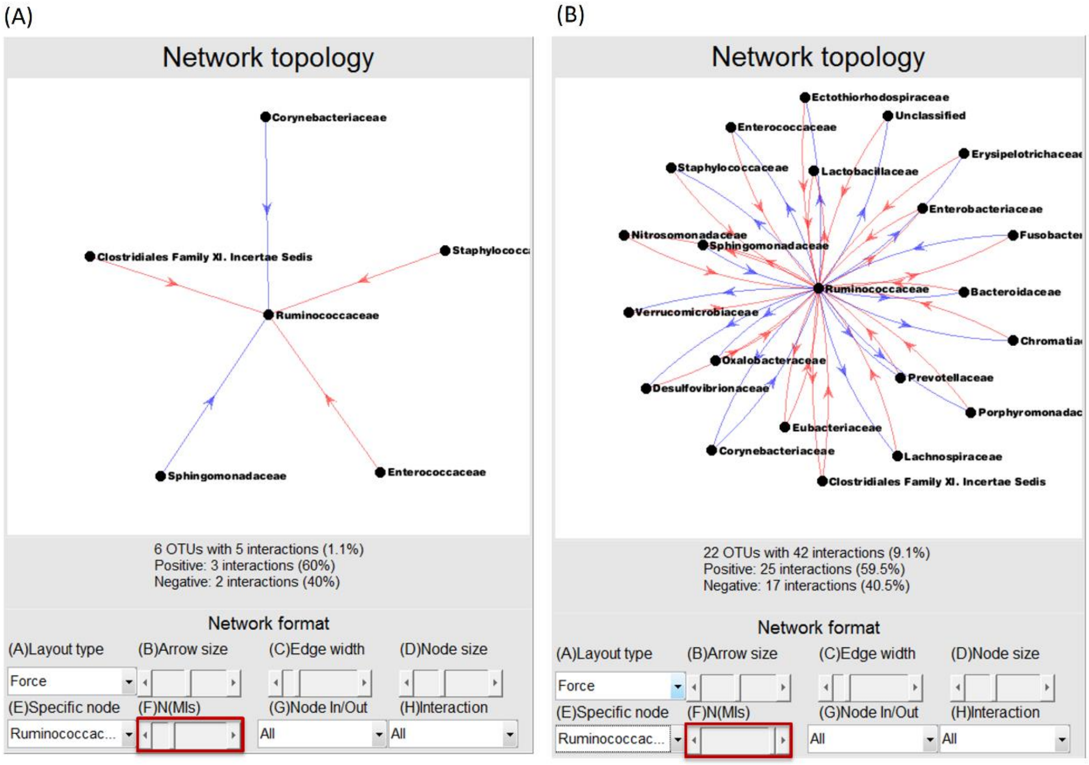

Figure 9 The interactive behaviors of a specific OTU to all others, exemplified by *Ruminococcaceae*. (A) Display fewer interactions with stronger strengths. (B) Display all interactions connected with *Ruminococcaceae*.

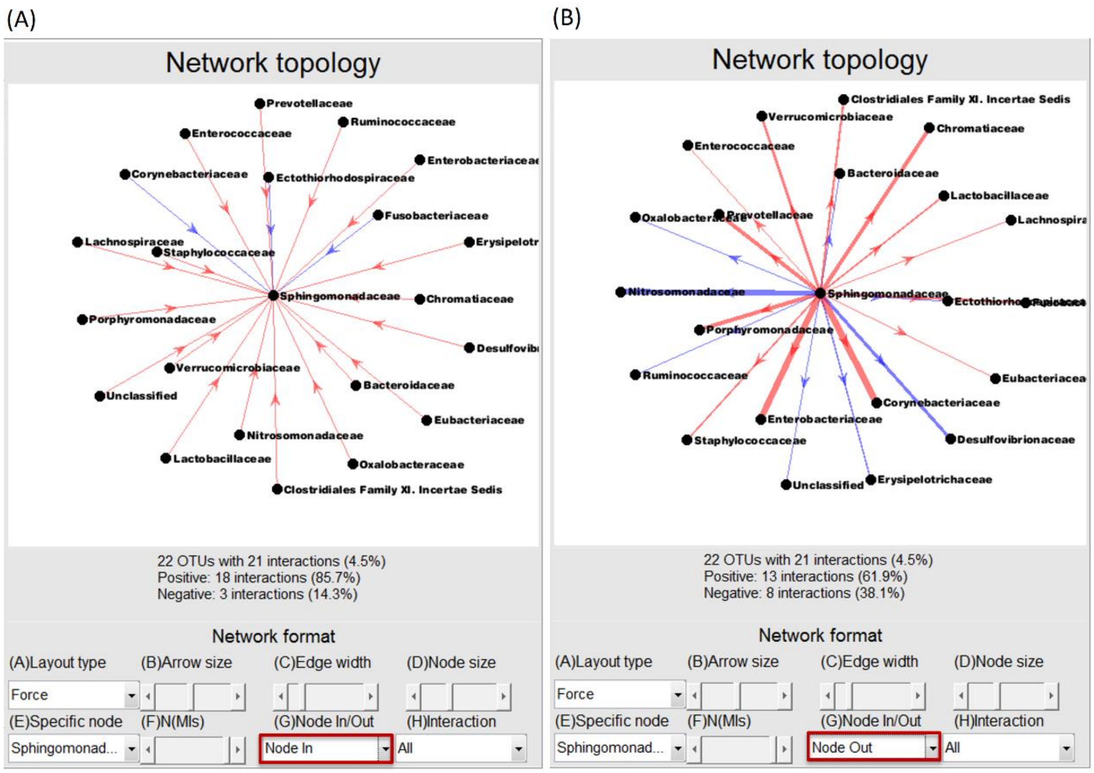

Figure 10 The graphic view of (A) Node In or (B) Node Out. This example was taken by *Sphingomonadaceae* while all interaction relations were selected.

.

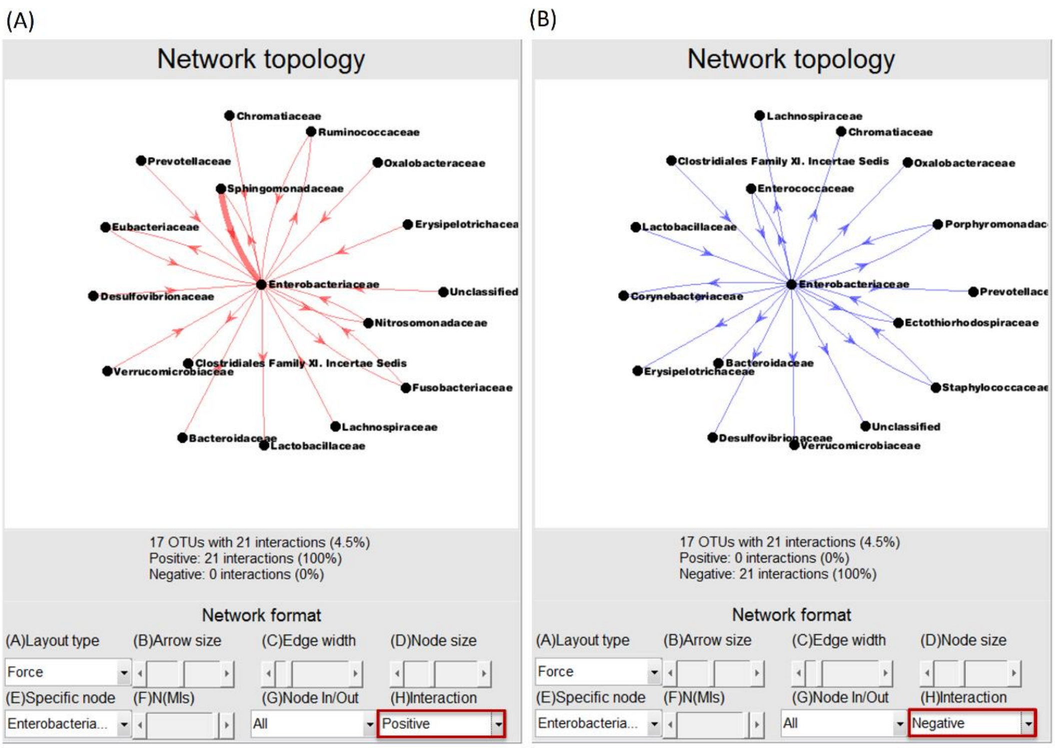

Figure 11 The network topology contains only (A) positive or (B) negative interactions, exemplified by *Enterococcaceae*.

6.1.3. Panel of "Overview of interaction pairs"

The main purpose of this panel is to identify potential key OTUs by observing the interactive behaviors between one OTU and others. If there are N OTUs in an interaction network, each OTU will have at most N-1 interaction-pair relationships. The distribution of six interaction patterns, including mutualism (+/+), competition (-/-), parasitism/predation (+/-), commensalism (+/0), amensalism (-/0), and no effect (0/0), is provided for each OTU (Fig. 12(A)). Users can fine-tune the threshold by turning weaker interactions to zero to reveal the influence of weaker interactions on the total distribution of interaction pairs (Fig. 12(B)). The average or summation of all absolute interaction strength from each interaction pattern is illustrated in Fig. 12(C). The two-dimensional principal component analysis (PCA) plot is supported to identify the major interaction pairs of an OTU (Fig. 12(D)).

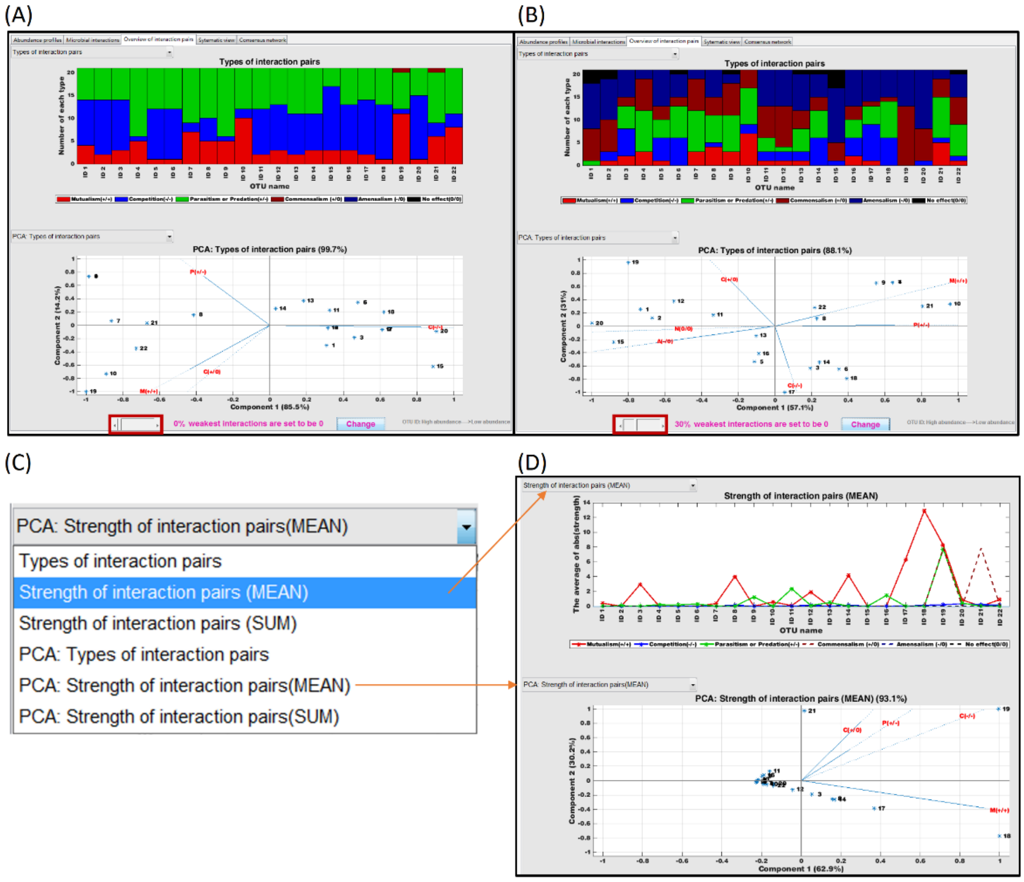

Figure 12 Panel of "Overview of interaction pairs" shows the different types of interaction pairs. PCA plot is supported to reveal the key OTUs among interaction patterns.

6.1.4 Panel of “Systematic view”

To compare outputs from different interaction networks, a systematic perspective is shown in Fig. 13. The distribution of three interaction patterns, including mutualism (+/+), competition (-/-), and parasitism/predation (+/-), is shown in Figure 11. The meaning of symbols is illustrated bellow.

O: parasitism or predation (+/-)

O: competition (-/-)

O: mutualism (+/+)

*: Interaction networks with successful outcomes are denoted as black star

*: Interaction networks with failed outcomes are denoted as gray star

For any two sequential interaction networks, one OTU may play a critical role in the system. The removal or addition of this lowest abundance OTU may influence the successful or failed outcomes. This column of “Status change” is used to record the status change between two sequential interaction networks (Table 1).

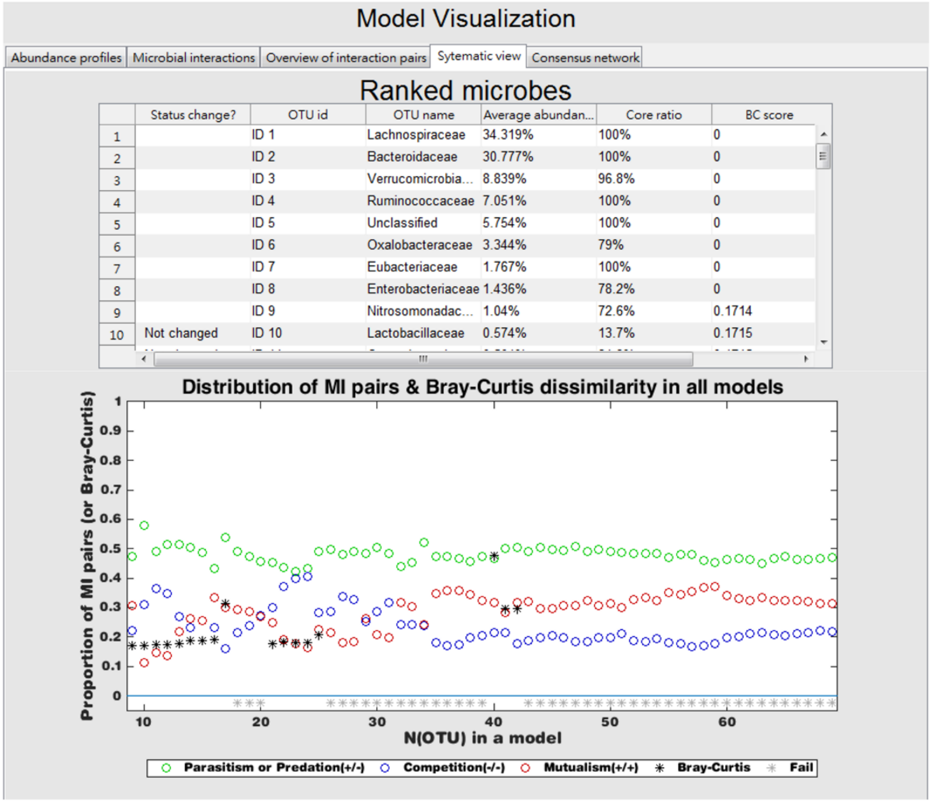

	Status change?	OTU id	OTU name	Average abundan...	Core ratio	BC score
1		ID 1	Lachnospiraceae	34.319%	100%	0
2		ID 2	Bacteroidaceae	30.777%	100%	0
3		ID 3	Verrucomicrobia...	8.839%	96.8%	0
4		ID 4	Ruminococcaceae	7.051%	100%	0
5		ID 5	Unclassified	5.754%	100%	0
6		ID 6	Oxalobacteraceae	3.344%	79%	0
7		ID 7	Eubacteriaceae	1.767%	100%	0
8		ID 8	Enterobacteriaceae	1.436%	78.2%	0
9		ID 9	Nitrosomonadac...	1.04%	72.6%	0.1714
10	Not changed	ID 10	Lactobacillaceae	0.574%	13.7%	0.1715

Figure 13 The panel of “systematic view” to examine all interaction networks.

As exemplified in Table 2, *Lactobacillaceae* did not exist in 9-OTU interaction network but in 10-OTU interaction network. The two interaction networks were denoted as success conveying BC scores, 0.1714 and 0.1715 respectively. Consequently, *Lactobacillaceae* would be denoted as “Not changed”. On the other hand, *Porphyromonadaceae* was involved in 18-OTU interaction network but absent in 17-OTU interaction network. The previous one resulted in a successful outcome (BC=0.3141) but the latter was failed (BC=0). Consequently *Porphyromonadaceae* should be annotated as “Status change: failure -> success”.

Table 1 The status change of two sequential interaction networks

**Status Change**	**Network A** (with $OTU_{lowest\ abundance}$)	**Network B** (without $OTU_{lowest\ abundance}$)
Not changed	Success	Success
Not changed	Failure	Failure
(Status change: success -> failure)	Success	Failure
(Status change: failure -> success)	Failure	Success

Table 2 The status change of interaction networks from female fecal microbiome.

**Status Change?**	**OTU ID**	**OTU Name**	**BC score**
	ID 9	*Nitrosomonadaceae*	0.1714
Not changed	ID 10	***Lactobacillaceae***	0.1715
Not changed	ID 11	*Corynebacteriaceae*	0.1745
Not changed	ID 12	*Staphylococcaceae*	0.1724
Not changed	ID 13	*Erysipelotrichaceae*	0.1782
Not changed	ID 14	*Prevotellaceae*	0.1862
Not changed	ID 15	*Ectothiorhodospiraceae*	0.1863
Not changed	ID 16	*Desulfovibrionaceae*	0.1907
Not changed	ID 17	*Chromatiaceae*	0.3141
(Status change: failure -> success)	ID 18	***Porphyromonadaceae***	0
Not changed	ID 19	*Sphingomonadaceae*	0
Not changed	ID 20	*Enterococcaceae*	0
(Status change: success -> failure)	ID 21	***Fusobacteriaceae***	0.1757

6.1.5 Panel of "Consensus network"

The panel of "Consensus network" is aimed at providing consensus interactions from all interaction networks (Fig. 14). For an interaction pair, Mij, there were n_{ij}^{+} and n_{ij}^{-} interaction networks producing positive and negative outcomes when the interactive direction was fixed. When the ratio of n_{ij}^{+} to the summation of n_{ij}^{+} and n_{ij}^{-} was statistically significantly greater than the user-defined threshold for this study, i.e., 90% represented by P(concordant pairs)>=0.9 in this panel, we were able to conclude that this interaction relation was concordant among networks and directed positively, and vice versa. One sample z-test for proportions was used to measure the concordance of predicted interactive relations among networks.

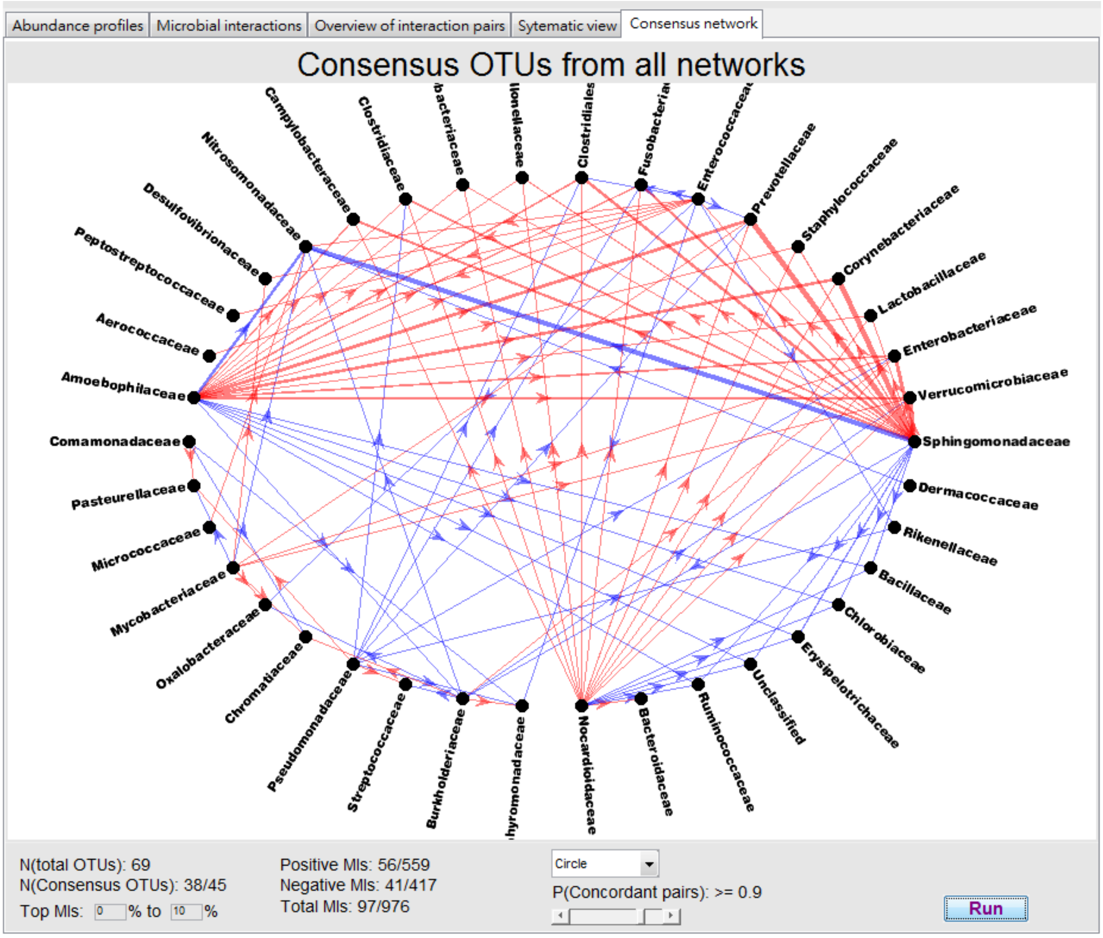

Figure 14 The panel of "Consensus network" can reveal consensus interactions from all interaction networks. The exemplified case was set by default setting.

There are three optional items to adjust the setting in the panel of “Consensus network” and listed below.

(A) 「Top MIs」:

This item is used to extract an interaction subnetwork with a specific range of interaction strengths. There are two columns to specify the range of interaction strengths.

For example, if we intended to display the strongest 10% of interactions, the upper and lower bound should be “0” % and “10” % (Fig. 14). On the contrary, the weakest 10% of interactions are extracted by the settings of “90” % and “100” % (Fig. 15).

(B) 「Layout」:

Similar to the layout options in the panel of “Microbial interactions”, there are four options, including circle, force, layered, or subspace, in this item.

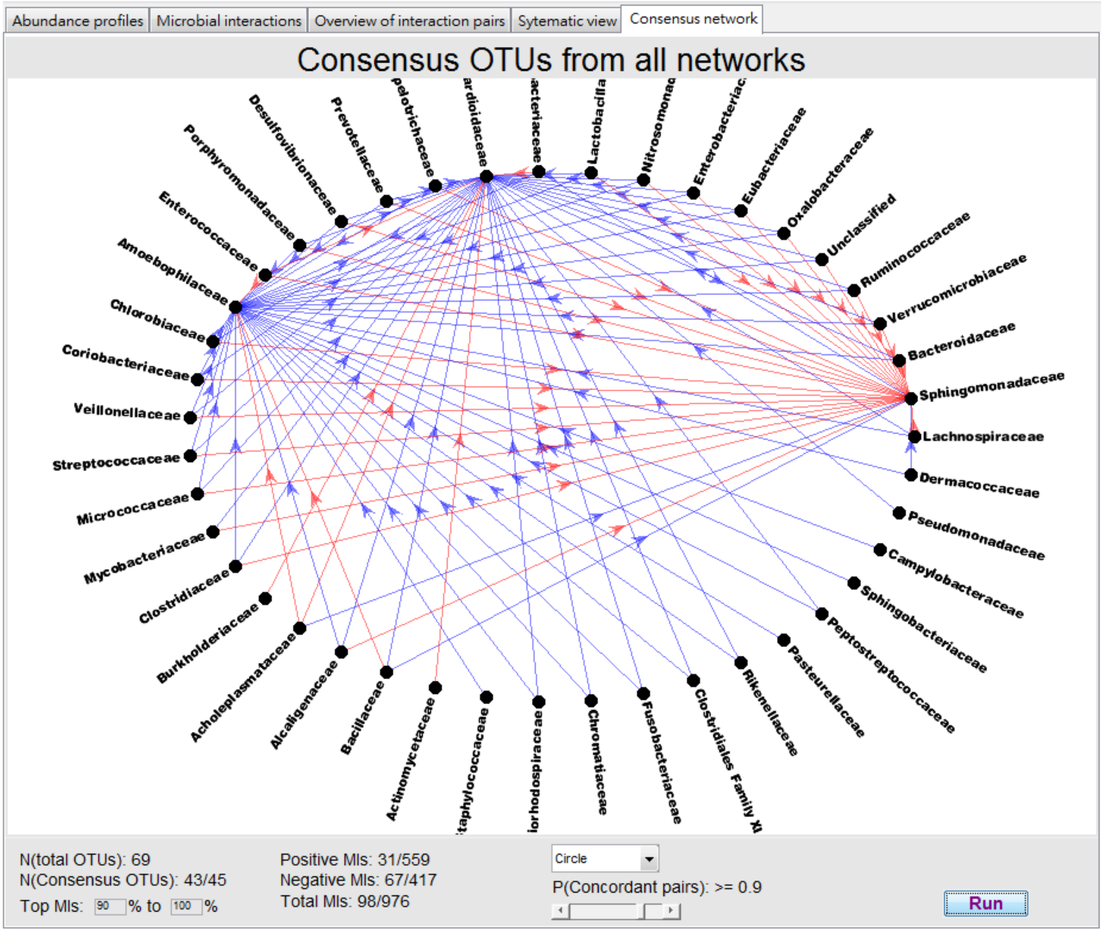

Figure 15 The optional item of “Top MIs” was set to extract the weakest 10% of interaction strengths.

(C) 「P(concordant pairs)」:

P(concordant pairs) is a threshold to determine the interaction consistency from multiple interaction networks. For example, P(concordant pairs)>=0.9 means to collect a set of interaction pairs, usually a source and a target OTUs, with 90% of interactive outcomes being consistent (Fig. 14). If the threshold is raised, fewer interactions are selected (Fig. 16).

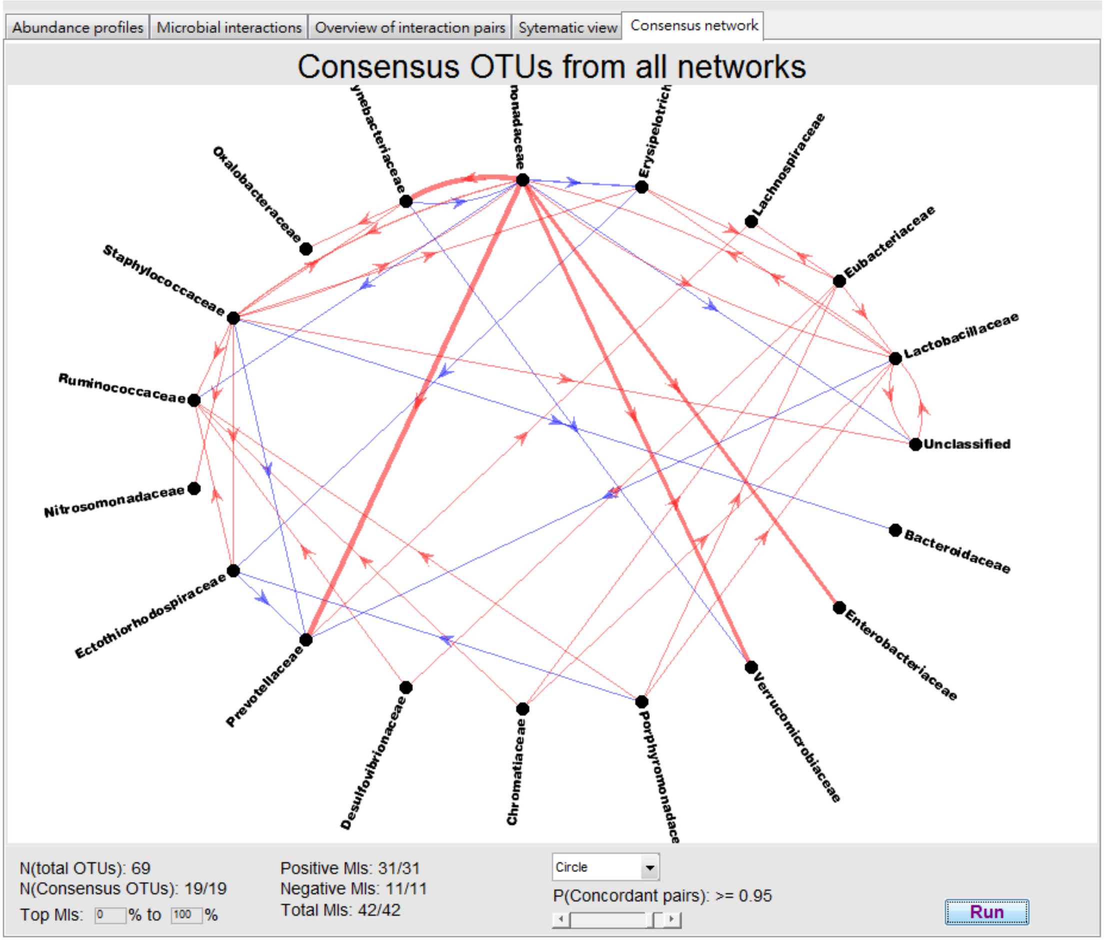

Figure 16 The optional item of "P(concordant pairs)" was set to 0.95 to increase the criteria of selecting consensus interactions.

6.2 Export File

6.2.1 Save to a mat file

After the implement of MetaMIS, all results and parameter settings can be saved into a .mat file, which is 「Save」on the left side of MetaMIS (Fig. 2). This .mat file can be loaded directly into the MetaMIS via the button of 「Open」.

6.2.2 Export interaction tables

Exporting the inferred microbial interaction table is an easy step. Choose 「This table」 to export the selected interaction table. Choose 「All tables」 to export all interaction tables (Fig .6). There are two kinds of tab separated files to produce. Filename_EDGE_N(OTUs).txt storages the inferred microbial interaction table, e.g. F_gut_EDGE_9.txt. Each OTU identifier has an average abundance value and a core ratio across samples, been reserved in the file of Filename_NODE_N(OTUs).txt, e.g. F_gut_NODE_9.txt.

6.2.3 How to import an interaction table to Gephi [4]?

Step 1: Double click the icon

Step 2: Create a new project (Fig. 17)

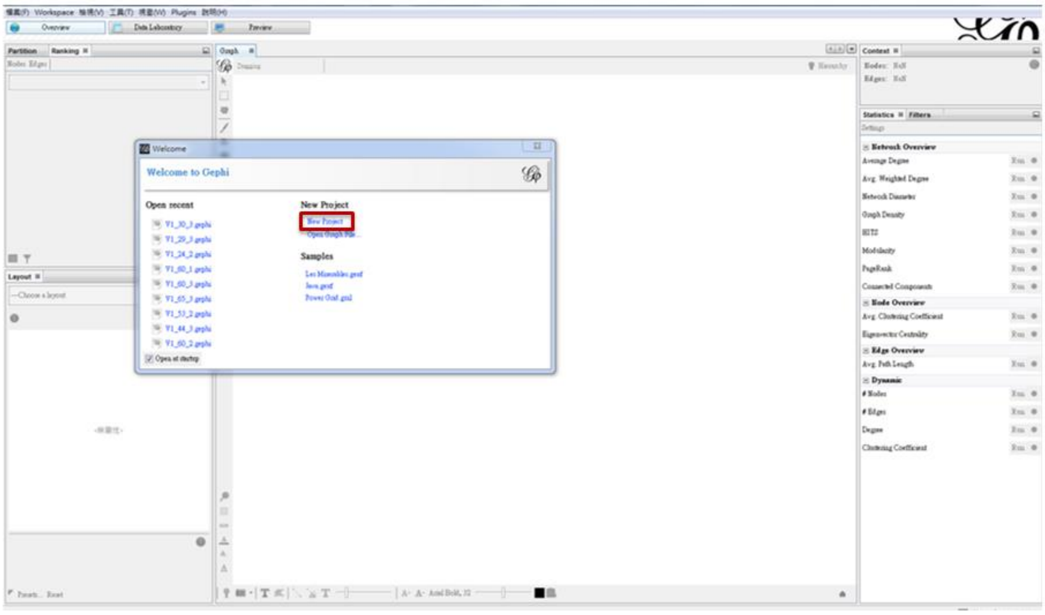

Figure 17 Create a new project in Gephi.

Step 3: To import the interactions, please follow the indicators in the Fig. 18

Step 4: Users can follow similar steps to import F_gut_NODE_9.txt, which provides OTU information about its average abundance and core ratio among samples. OTU information may help users to understand the topological network.

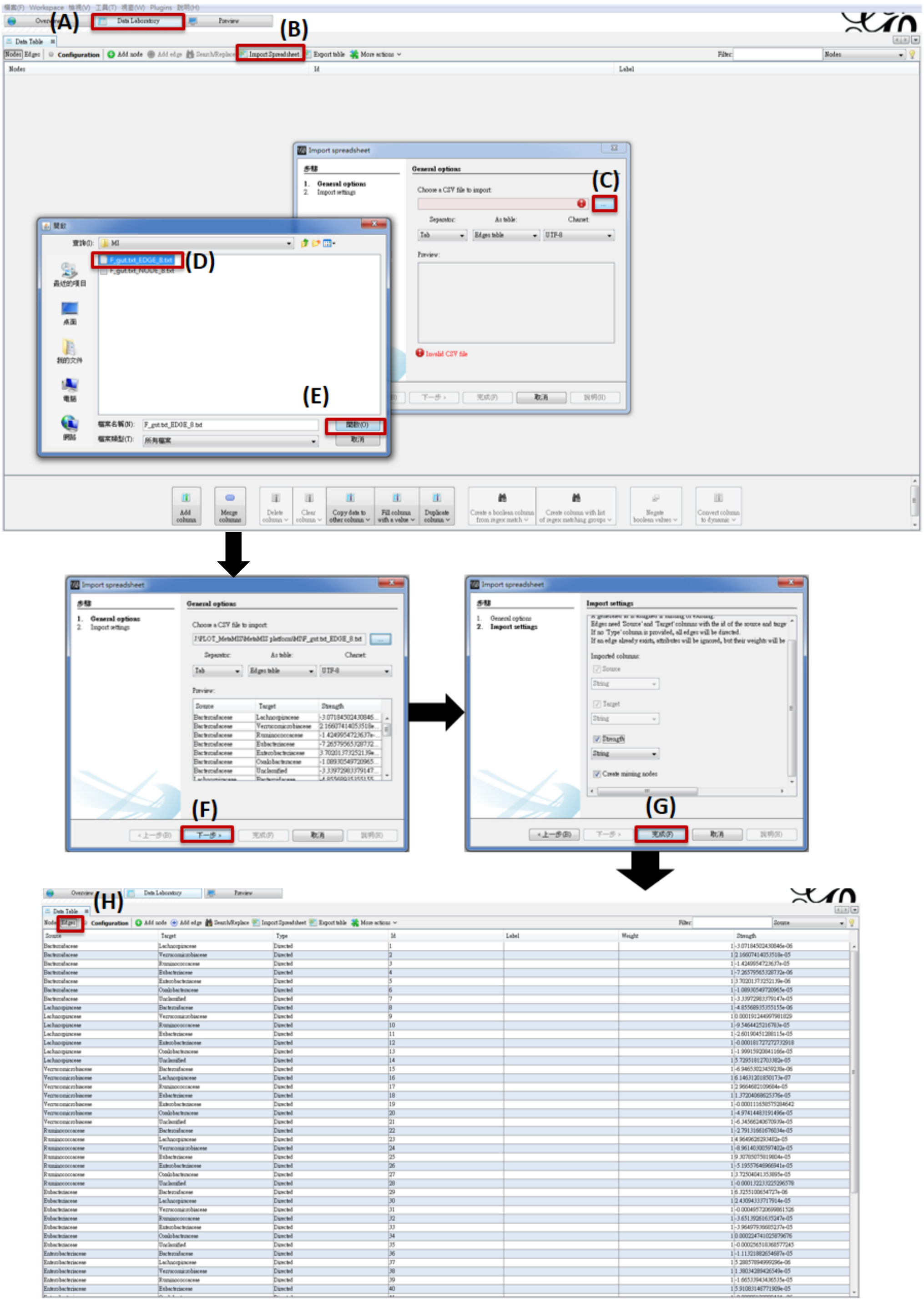

Figure 18 The process of importing an interaction table into Gephi.

6.2.4 How to import an interaction table to Cytoscape [5]?

Step 1: Double click the icon 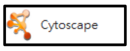

Step 2: Create a new project and follow the process in Figure 19.

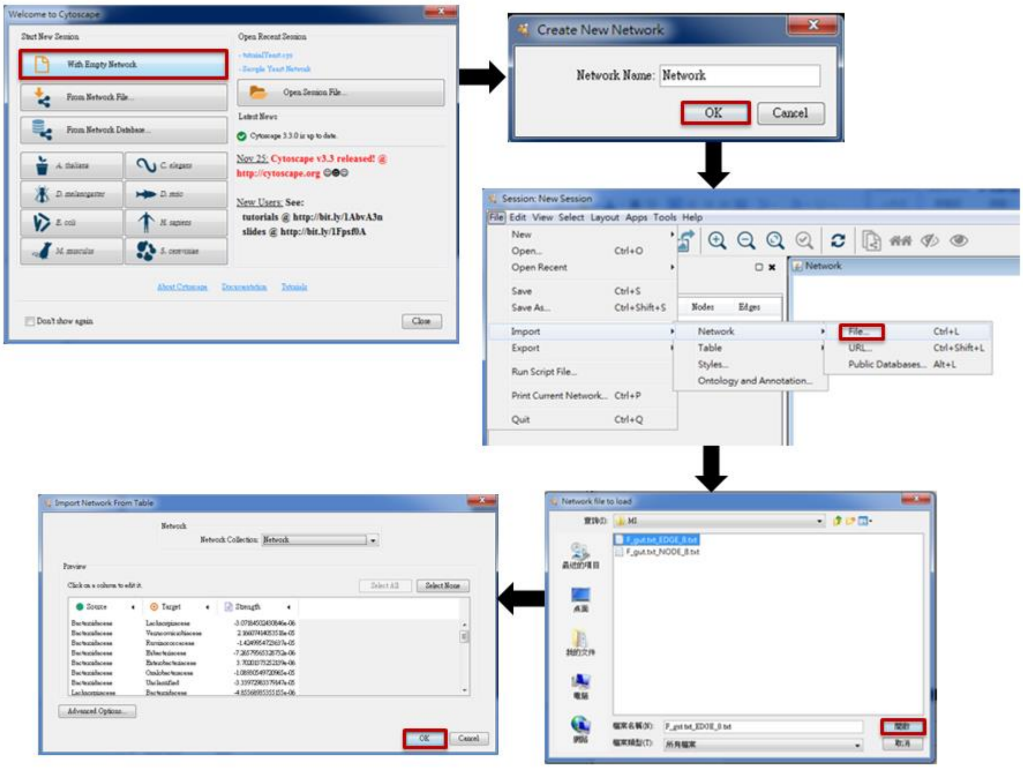

Figure 19 The process of importing an interaction table into Cytoscape.

Bugs or problems

Encounter bugs, problems, or have any suggestions? Please contact Grace Tzun-Wen Shaw (tzunwen@gmail.com)

References

1. Caporaso, J.G., et al., *Moving pictures of the human microbiome.* Genome Biol, 2011. **12**(5): p. R50.
2. Angly, F.E., et al., *CopyRighter: a rapid tool for improving the accuracy of microbial community profiles through lineage-specific gene copy number correction.* Microbiome, 2014. **2**.
3. Jansen, W., *A permanence theorem for replicator and Lotka-Volterra systems.* Journal of Mathematical Biology, 1987. **25**(4): p. 411-422.
4. Bastian, M., S. Heymann, and M. Jacomy, *Gephi: an open source software for exploring and manipulating networks.* ICWSM, 2009. **8**: p. 361-362.
5. Shannon, P., et al., *Cytoscape: A software environment for integrated models of biomolecular interaction networks.* Genome Res, 2003. **13**(11): p. 2498-2504.
